# Cytotoxic naphtho- and benzofurans from an endophytic fungus *Epicoccum nigrum* Ann-B-2 associated with *Annona squamosa* fruits

**DOI:** 10.1038/s41598-024-55168-5

**Published:** 2024-02-28

**Authors:** Mohamed S. Elnaggar, Shaimaa Fayez, Alaa Anwar, Sherif S. Ebada

**Affiliations:** Department of Pharmacognosy, Faculty of Pharmacy, Ain Shams University, Cairo, 11566 Egypt

**Keywords:** Cancer, Drug discovery, Chemistry

## Abstract

Chemical exploration of the total extract derived from *Epicoccum nigrum* Ann-B-2, an endophyte associated with *Annona squamosa* fruits, afforded two new metabolites, epicoccofuran A (**1**) and flavimycin C (**2**), along with four known compounds namely, epicocconigrone A (**3**), epicoccolide B (**4**), epicoccone (**5**) and 4,5,6-trihydroxy-7-methyl-1,3-dihydroisobenzofuran (**6**). Structures of the isolated compounds were elucidated using extensive 1D and 2D NMR along with HR-ESI–MS. Flavimycin C (**2**) was isolated as an epimeric mixture of its two diastereomers **2a** and **2b**. The new compounds **1** and **2** displayed moderate activity against *B. subtilis*, whereas compounds (**2**, **3**, **5,** and **6**) showed significant antiproliferative effects against a panel of seven different cancer cell lines with IC_50_ values ranging from 1.3 to 12 µM.

## Introduction

Literally speaking, the word endophytes means "inside or within the plant" which is a broad definition extending the spectrum of plant inhabitants to bacteria, fungi, and even insects that can colonize any organ in the plant either facultative or obligatory where their symbiotic association is, in most circumstances, harmless to the host plant^1,2^. While endophytic bacteria can reside inside or on the surface of disinfected plant tissues, endophytic fungi generally reside inside the tissues without causing any signs of overt harm to the host plant^1^. Quorum sensing is a cell-to-cell communication system that allows endophytes to overcome the host immune system^3,4^. Over the past decades, numerous benefits have been reported for endophytes including the biological control of phytopathogens^5–7^, enhancement of plant defense and growth^2,8–10^, environmental clean-up operations^11^, industrial purposes^11,12^, and the production of metabolites with great biotechnological, agricultural^13,14^, and pharmaceutical relevance^3,9,15–17^. Fungal endophytes have been found in almost all plant families around the Globe and adapting to all climatic conditions^3^. They have long been a rich reservoir of pharmacologically active metabolites that served as lead structures for the development of new antimicrobial and anticancer drugs^3,15,18–21^ specially after the evolution of resistance and unresponsiveness to the current in-use chemotherapeutics^22,23^. The genus *Epicoccum *was reported from several plant or marine organisms and is used in the biocontrol of many pathogenic fungi due to its ability to produce a vast array of secondary metabolites including polyketides^24,25^, diketopiperazines^26–28^, carotenoids^29^, siderophores^30^, alkaloids^31^, glycosylated tetramic acid derivatives^32^, and depsipeptides^33,34^ with reported antimicrobial and cytotoxic effects^35^. The widespread mitosporic ascomycete *Epicoccum nigrum* can colonize different soils and host organisms. Studies showed that *E. nigrum* had the potential to produce the commercial fluorophore "epicocconone" which is used in cell staining and gel electrophoresis^35^. The preharvest application of *E. nigrum* showed efficiency in controlling brown rot disease in peaches^36,37^ and potato late blight in potatoes^38^. Pigments produced from *E. nigrum* are safe and non-toxic making them of potential use in industry^35^. Diverse compounds have been reported from *E. nigrum* specially with antimicrobial and cytotoxic activities^39–43^. In the course of our search for antimicrobial and antitumor compounds from plant-associated fungi, we report in this study the isolation and structure elucidation of two previously undescribed naphtho- and benzofuran derivatives (**1** and **2**) along with four known metabolites, epicocconigrone A (**3**)^40^, epicoccolide B (**4**)^24^, epicoccone (**5**)^44,45^, and 4,5,6-trihydroxy-7-methyl-1,3-dihydroisobenzofuran (**6**)^45,46^, from the total extract of *Epicoccum nigrum* Ann-B-2 derived from *Annona squamosa* fruits together with their results in antimicrobial and cytotoxicity assays.

## Materials and methods

### General experimental procedures

Optical rotation values were determined on an Anton Paar MCP-150 polarimeter (Seelze, Germany) at room temperature in DMSO or methanol at 589 nm and 100 mm path length. The NMR data (1D and 2D) were acquired in DMSO-*d*_*6*_ on a Bruker Avance III 500 spectrometer (Bremen, Germany) equipped with a 5 mm TXI cryoprobe (^1^H NMR 500 MHz and ^13^C NMR 125 MHz) or Avance III 700 (Bruker, ^1^H 700 MHz, ^13^C 175 MHz) spectrometers referenced to the residual solvent peak 2.50 ppm and 39.52 ppm for ^1^H and ^13^C NMR, respectively. The HR-ESI–MS analysis were conducted on a maXis ESI-TOF mass spectrometer with a capillary voltage set at 4500 V and a scan range covering *m/z* 100–2500. The mass spectrometer was connected to an Agilent 1200 HPLC–UV system (Santa Clara, CA, USA) scanning over 200–400 nm. Molecular formulas of the indicated compounds were calculated using the Smart Formula algorithm of the Compass Data Analysis software (Bruker, version 4.4). Analytical HPLC was performed using a Dionex UltiMate 3000 UHPLC (Thermo Fisher Scientific Inc.) equipped with a C18 Acquity UPLC BEH column (2.1 × 50 mm, 1.7 µm, Waters, Milford, USA) and the UV/Vis detections recorded at 190–600 nm. The following gradient was implemented as a mobile phase: 5% B for 0.5 min reaching 100% B in 19.5 min with a flow rate at 0.6 ml/min where solvent A is water + 0.1% formic acid (v/v) and solvent B is acetonitrile + 0.1% formic acid (v/v). The HPLC solvents were purchased from Merck Co. (Darmstadt, Germany).

### Fungal material

*Epicoccum nigrum* Ann-B-2 strain was isolated and purified from the inner tissues of the edible fruit *Anonna squamosa.* Annona fruits were collected from different locations within Cairo Governorate, Egypt, in February 2021, and was thankfully authenticated by taxonomist Dr. Terraize Demian. The collection was complied with the IUCN Policy Statement on Research Involving Species at Risk of extinction and collection requirements and were carefully followed in the conduct of this research to comply with institutional, national, and international guidelines and legislation. To isolate the fungal strain, the edible fruit was rinsed with distilled water then surface sterilization was performed using 70% ethanol for 60 s. Small pieces from the innermost tissues of the edible fruit were aseptically cut using sterilized blade and pressed onto malt extract agar plate (15 g/L malt extract, 15 g/L agar, 0.2 g/L chloramphenicol to suppress bacterial growth, pH adjusted to 7.4–7.8 using 10% NaOH if needed). After incubation at 25°C, inspection for the fungal growth was followed in which the fungal strain under investigation was found to grow out from the fruit tissues. Pure fungal strains were grown by repeated re-inoculation on fresh culture media.

### Fermentation and extraction

Fermentation of fungal strain was performed on solid rice culture media (100 g rice in 110 mL distilled water, autoclaved for 20 min at 121 °C) in 1L Erlenmeyer flask (12 flasks were used). The culture was incubated for 6 weeks at 25 °C under static conditions. After the fermentation period, fungal biomass were extracted successively with ethyl acetate (3 × 600 mL per flask), combined, and concentrated under vacuum where the residue was defatted between *n*-hexane and 90% aqueous methanol to give a total of 6.0 g dark brown extract by evaporating the aqueous phase under reduced pressure.

### DNA extraction and PCR analysis

DNA amplification and sequencing of the ITS region was implemented using a similar protocol as previously described by Kjer et al.^47^. The sequence was submitted to GenBank for comparison leading to a 99% identity to *Epicoccum nigrum* Ann-B-2 with an accession code OQ780784.

### Isolation of compounds 1–6

The defatted methanol-soluble extract (6.0 g) was fractionated on silica using vacuum liquid chromatography by implementing a gradient system composed of *n*-heptane:dichloromethane (10:0, 7:3, 3:7, and 0:10) then another gradient formed from dichloromethane:methanol at 10:0, 9:1, 3:2, and 0:10. After pooling similar fractions based on their LCMS dereplication, a total of eight fractions (E_1_–E_8_) were obtained. Fraction E_6_ (940 mg, eluted from DCM:MeOH (9:1)) revealed unprecedented masses in LCMS and was further fractionated on 50 g of Diaion HP20 using a gradient system of water:acetonitrile (10:0, 7:3, 1:1, 3:7, and 0:10) yielding five subfractions (E_6_H_1_–E_6_H_5_). Subfraction E_6_H_2_ (80 mg) was purified on a preparative HPLC (PLC 2020; Gilson) using Gemini C_18_ (250 mm × 21 mm, 10 μm: Phenomenex) column, solvent A (deionized H_2_O + 0.1% TFA (v/v)) and solvent B (acetonitrile + 0.1% TFA (v/v)); a flow rate of 15 mL/min; and the UV detection at 210, 254 and 280 nm; gradient: 5 min holding at 5% B, increasing B to 25% in 15 min, increasing B to 100% B in 25 min, and then holding at 100% B for 10 min) to yield compounds **6** (2.5 mg, *t*_R_ = 17 min) and **5** (1.5 mg, *t*_R_ = 22 min). Using the same preparative HPLC conditions, subfraction E_6_H_5_ (190 mg) was purified to afford compounds **2** (2.0 mg, *t*_R_ = 27 min), **1** (2.0 mg, *t*_R_ = 28 min), **3** (3.0 mg, *t*_R_ = 29 min), and **4** (3.0 mg, *t*_R_ = 31 min).

#### Epicoccofuran A (*1*)

Off-white amorphous powder; UV (MeOH) λ_max_ 203, 222, 308, and 437 nm; ^1^H and ^13^C NMR see Table 1; in LR-ESI-MS *m/z* 291.0 and at *m/z* 289.0; HR-ESI–MS *m/z* 291.0859 [M + H]^+^ (calcd. C_15_H_15_O_6_^+^; 291.0858).

**Table 1:** 1D (^1^H and ^13^C) and 2D (HMBC and ROESY) NMR data of epicoccofuran A (**1**).

Pos	*δ*_C_,^a,c^ type	*δ*_H_^b^ (*J* in Hz)	HMBC^b^	ROESY^b^
1	78.2, CH_2_	5.13 (t, 3.1, 2H)	3w,^d^ 4w,^d^ 12w,^d^ 13w^d^	H_3_-15
3	75.5, CH_2_	4.97 (t, 3.1, 2H)	1w,^d^ 4w,^d^ 5w,^d^ 13w^d^	H_3_-14
4	134.5, C			
5	119.9, C			
6	151.0, C			
7	132.7, C			
8	149.0, C	14.89 (brs, O*H*)		
9	116.8, C			
10	150.7, C			
11	114.2, C			
12	127.3, C			
13	133.0, C			
14	14.9, CH_3_	2.09 (s, 3H)	4, 5, 6	H_2_-3
15	15.2, CH_3_	2.19 (s, 3H)	10, 11, 12	H_2_-1
16	183.4, CO			

Measured in DMSO-*d*_*6*_^a^at 125 MHz / ^b^at 500 MHz.

^c^Assigned based on HMBC and HSQC spectra.

^d^ ”w” denotes a weak correlation.

#### Flavimycin C (*2*)

Pale yellow powder; $$\left[\alpha \right]\begin{array}{c}20\\ D\end{array}$$ +2.1 (*c* 0.2, DMSO); UV (MeOH) λ_max_ 210 and 270 nm; ^1^H and ^13^C NMR see Table 2; in LRESIMS *m/z* 361.0 and *m/z* 359.0; HR-ESI–MS *m/z* 361.0917 [M-CH_3_OH + H]^+^ (calcd. C_18_H_17_O_8_^+^; 361.0919) and *m/z* 359.0771 [M-CH_3_OH-H]^−^ (calcd. C_18_H_15_O_8_^−^; 359.0768).

**Table 2:** 1D (^1^H and ^13^C) NMR data of flavimycin C (**2a** and **2b**).

Pos	2a		2b	
	*δ*_C_,^a,c^ type	*δ*_H_^b^ (*J* in Hz)	*δ*_C_,^a,c^ type	*δ*_H_^b^ (*J* in Hz)
1	108.0, CH	6.18 (s, 1H)	106.6, CH	6.24 (d, 2.2, 1H)
3	84.5, CH	5.10 (d, 8.3, 1H)	85.0, CH	5.35, (dd, 7.0, 2.2, 1H)
4	116.6, C		116.9, C	
5	137.7, C		137.6, C	
6	134.3, C		134.2, C	
7	145.0, C		144.9, C	
8	110.8, C		110.5, C	
9	125.8, C		126.2, C	
10	11.1, CH_3_	2.00 (s, 3H)	11.1, CH_3_	1.99 (s, 3H)
11	54.2, CH_3_	3.37 (s, 3H)	52.0, CH_3_	3.12 (s, 3H)
1′	72.8, CH_2_	*α* 4.93 (d, 11.0, 1H)	72.9, CH_2_	*α* 4.96 (d, 10.4, 1H)
		*β* 5.10 (dd, 11.0, 2.5, 1H)		*β* 5.22 (dd, 10.4, 5.1, 1H)
3′	87.2, CH	5.20 (m, overlapped, 1H)	86.4, CH	5.21 (m, overlapped, 1H)
4′	114.5, C		114.8, C	
5′	138.5, C		138.2, C	
6′	132.3, C		132.5, C	
7′	144.8, C		145.0, C	
8′	108.2, C		108.4, C	
9′	128.5, C		128.3, C	
10′	12.1, CH_3_	1.92 (s, 3H)	12.1, CH_3_	1.94 (s, 3H)

Measured in DMSO-*d*_*6*_^a^at 175 MHz / ^b^at 700 MHz.

^c^Assignment confirmed by HMBC and HSQC spectra.

### Antimicrobial assay

A panel of Gram-positive and Gram-negative bacteria along with fungal strains (Table 3), namely; (bacteria: *Staphylococcus aureus* [DSM 346], *Mycobacterium smegmatis* [ATCC 700084] and *Bacillus subtilis* [DSM 10] and fungi: *Mucor hiemalis* [DSM 2656], *Candida albicans* [DSM 1665] and *Rhodotorula glutinis* [DSM 10134]) were used to evaluate the minimum inhibitory concentrations (MIC) of the isolated compounds **1**–**6** using a 96-well plate serial dilution assay over a concentration range from 67 to 0.5 µg/mL as described in our previous work^47,48^.

**Table 3:** The minimum inhibitory concentrations (MIC) of compounds **1**–**6**.

Tested organism	MIC (µg/ml)						
	1	2	3	4	5	6	Standard*
*Staphylococcus aureus*	> 67	> 67	> 67	> 67	> 67	> 67	0.21^G^
*Mycobacterium smegmatis*	> 67	> 67	> 67	> 67	> 67	> 67	1.7^ K^
*Bacillus subtilis*	33.3	33.3	66.6	66.6	> 67	> 67	16.6^O^
*Mucor hiemalis*	> 67	> 67	> 67	> 67	> 67	> 67	2.1^N^
*Candida albicans*	> 67	> 67	> 67	> 67	> 67	> 67	2.1^N^
*Rhodotorula glutinis*	> 67	> 67	> 67	> 67	> 67	> 67	2.1^N^

^G^Gentamycin 1 mg/mL, ^K^Kanamycin 1 mg/mL, ^O^Oxytetracycline 1 mg/mL, ^N^Nystatin 10 mg/mL.

### Cytotoxicity assay

In vitro cytotoxicity studies (data expressed in the form of IC_50_ values) were performed over a concentration range between 1 and 37 µg/mL on a panel of seven cancer cell lines including human endocervical adenocarcinoma (KB3.1), mouse fibroblasts (L929), lung cancer (A549), squamous cancer (A431), prostate cancer (PC-3), breast cancer (MCF-7), and ovary cancer (SK-OV-3) using the MTT assay as previously reported^49^.

## Results and discussion

**Figure 1:**
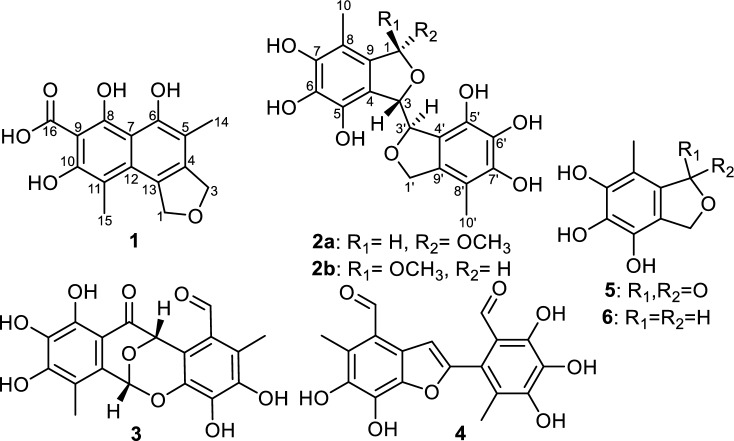
Chemical structures of **1**–**6**.

In this study, *Epicoccum nigrum* Ann-B-2 fungus was isolated as an endophyte associated with *A. squamosa* fruits. The fungus was fermented on rice medium over four weeks at 25 °C under static conditions then extracted with ethyl acetate. The total extract was concentrated and fractionated on VLC (vacuum liquid chromatography using silica gel as a stationary phase) to yield six compounds (Fig. 1), two of them (**1** and **2**) were recognized as previously undescribed natural products.

Compound **1** was isolated as an off-white amorphous powder with UV absorption maxima (*λ*_max_) at 203, 222, 308, and 437 nm. The molecular formula of **1** was established as C_15_H_14_O_6_ based on its HR-ESI–MS that revealed a protonated molecule at *m/z* 291.0859 [M + H]^+^ (calculated 291.0858) indicating nine degrees of unsaturation. The ^13^C NMR data (Table 1) and HSQC spectrum (Fig. S6) of **1** revealed fifteen carbon resonances distinguished into eleven unprotonated carbons including one carboxycarbonyl carbon at δ_c_ 183.4, three oxygenated olefinic carbons (δ_c_ 151.0, 150.7, and 149.0) and seven olefinic carbons (δ_c_ 134.5, 133.0, 132.7, 127.3, 119.9, 116.8, and 114.2) together with two deshielded oxymethylene carbons (δ_c_ 78.2 and 75.5) suggested to form a tetrahydrofuran ring and two aromatic methyl groups (δ_c_ 15.2 and 14.9).

**Figure 2:**
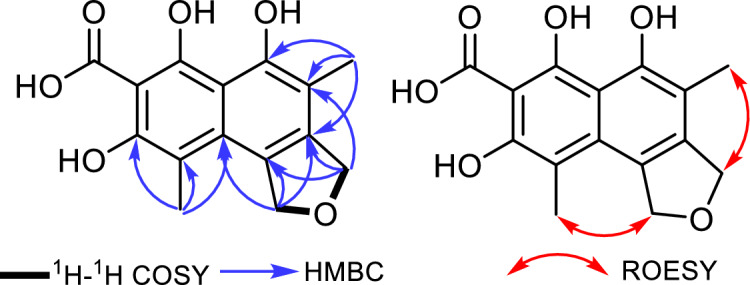
Key COSY, HMBC, and ROESY correlations of epicoccofuran A (**1**).

From the ^13^C NMR data of **1**, it was deduced to possess a tricyclic structure including one tetrahydrofuran ring and two fused aromatic rings as a naphthalene moiety comprising five double bonds interpreting eight degrees of unsaturation alongside with the carboxycarbonyl group. The ^1^H NMR data and HSQC spectrum of **1** (Table 1, Fig. S6) supported its suggested structural features by exhibiting two aromatic methyl singlets at δ_H_ 2.09 (Me-14; δ_C_ 14.9) and at δ_H_ 2.19 (Me-15; δ_C_ 15.2). The HMBC of **1** (Fig. 2) disclosed key correlations from oxymethylene protons at δ_H_ 5.13 (H_2_-1) and δ_H_ 4.97 (H_2_-3) to both quaternary olefinic carbon atoms at δ_C_ 134.5 (C-4) and δ_C_ 133.0 (C-13) ppm confirming their existence as a tetrahydrofuran ring directly fused to an aromatic ring.

The two aromatic methyl groups (Me-14 and Me-15) were situated at C-11 and C-5 of the naphthalene ring based on the ROESY spectrum (Fig. 2) exhibiting key ROE correlations to H_2_-3 and H_2_-1, respectively, together with the key HMBC correlations from Me-14 to C-4 and from Me-15 to C-10 (δ_C_ 150.7) and C-12 (δ_C_ 127.3). Based on the proposed structural characteristics and by searching the reported literature, compound **1** was found to be related to unusual dibenzofurans, preussifurans A-B, previously reported as antimalarial and moderate antiproliferative fungal metabolites from an endophytic fungus *Preussia* sp. isolated from the Cameronian medicinal plant *Enantia chlorantha* Oliv.^50^. However, the depicted structure of **1** (Fig. 1) revealed a tetrahydrofuran ring fused with a naphthalene moiety rather than being a dibenzofuran scaffold. Accordingly and to the best of our knowledge, compound **1** was identified as the first report of this naphthofuranoid skeleton in natural products and hence was given the trivial name epicoccofuran A.

**Figure 3:**
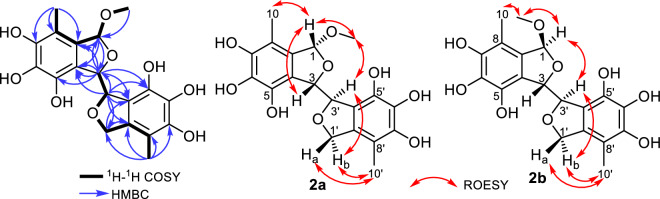
Key COSY, HMBC, and ROE correlations of flavimycin C (**2a** and **2b**).

Compound **2** was purified as a pale-yellow powder that revealed UV absorption maxima (*λ*_max_) at 210 and 270 nm. Its molecular formula was determined to be C_19_H_20_O_9_ indicating ten degrees of unsaturation based on HR-ESI–MS, ^1^H and ^13^C NMR data (Table 2, Figs. S9–S11). The ^1^H and ^13^C NMR spectra of **2** were recorded in DMSO-*d*_*6*_ and unveiled two sets of corresponding peaks in a ratio of 1:1 suggesting that **2** is an equal mixture of two epimers, **2a** and **2b**. Each of the two epimers comprised a singlet oxygenated methyl group at δ_H_ 3.37 and at δ_H_ 3.12 ppm that were correlated by HSQC to the two carbon resonances at δ_c_ 54.2 and 52.0 ppm, respectively. A literature search of **2** revealed its close resemblance to flavimycins A and B, previously reported from a soil-derived fungus *Aspergillus flavipes* by Kwon et al.^51^. By comparing the 1D and 2D NMR data (Table 2, Figs. 3 and S10–S14) of the two epimers **2a** and **2b** with those reported for flavimycin B^51^, it can be unambiguously determined that both epimers **2a** and **2b** feature its identical building units namely, 1,5,6,7-tetrahydroxy-8-methyl-1,3-dihydroisobenzofuran and 5,6,7-trihydroxy-8-methyl-1,3-dihydroisobenzofuran with a sole structural difference in the former through the presence of a methoxy group replacing the hydroxyl group at C-1. The methoxy groups were assigned at C-1 of the furan ring in each epimer as indicated by their HMBC correlations (Fig. 3) to two acetal carbon atoms resonating at δ_c_ 108.0 and δ_c_ 106.6 for the two epimers **2a** and **2b**, respectively.

The connection between the two isobenzofurans was established to be C3-C3′ as evidenced by the HMBC correlations from H-3 to C-4′ and C-5′ and from H-3′ to C-4. Two aromatic methyl groups assigned as Me-10′ at δ_H_ 1.92 and δ_H_ 1.94 disclosed key ROE correlations in ROESY spectrum (Fig. 3) to diastereotopic hydroxymethylene proton pairs ascribed to H_2_-1′ at δ_H_ 4.93/δ_H_ 5.10 (δ_c_ 72.8) and at δ_H_ 4.96/δ_H_ 5.22 (δ_c_ 72.8) in **2a** and **2b**, respectively, indicating their positions to be at C-8′ in each epimer.

The vicinal coupling (*J* = 8.3 and 7.0 Hz) between the two oxygenated methine protons at C-3 and C-3′ in **2a** and **2b**, respectively, suggested that both rings should be *trans*-configured. The relative configuration of the two methine protons at C-1 and C-3 in **2a** and **2b** were distinguished based on their ROE correlations where H-1 correlated to H-3 (in **2a**) and H-3′ (in **2b**) hinting at *cis* and *trans* configurations, respectively. Therefore, compound **2** was identified as a mixture of two epimers **2a**/**2b** and it was trivially named flavimycin C.

Among the tested strains, compounds **1** and **2** displayed moderate antimicrobial activities against *Bacillus subtilis* (Table 3)*,* however they showed no potential activity against the remaining strains. Their potency against *Bacillus* was almost half that of the standard oxytetracycline. For the cytotoxicity assessment, compounds **2** and **6** displayed the highest potency with significant inhibition to the proliferation of almost all cancer cell lines except for PC-3 (prostate cancer) which was insensitive (Table 4). Compounds **3** and **5** were likewise active against all tested cell lines, whereas compounds **1** and **4** showed weak anticancer activity.

**Table 4:** In vitro cytotoxicity assay results of compounds **1**–**6**.

Cancer cell line	IC_50_ (µM)						
	1	2	3	4	5	6	Epothilone B (nM)
KB3.1	16	7.8	2.7	21	6.1	3.4	0.17
L929	12	6.7	14	19	7.5	2.8	0.65
A549	n.t.^a^	9.2	10	n.t.^a^	6.8	6.3	0.05
A431	n.t.^a^	8.2	7.8	n.t.^a^	7.3	7.3	0.06
PC-3	n.t.^a^	> 100	8.0	n.t.^a^	8.0	> 100	0.09
MCF-7	n.t.^a^	2.7	2.4	n.t.^a^	3.8	1.3	0.07
SKOV-3	n.t.^a^	17	12	n.t.^a^	8.2	7.8	0.09

^a^n.t.: “not tested”.

## Conclusion

*Epicoccum* species produce diverse bioactive secondary metabolites, a reason for their common use as biocontrol agents against several phytopathogens. Systematic LC–MS-guided investigations for the culture extract and purified fractions of *E. nigrum* isolated from *Annona squamosa* plant resulted in the identification and full characterization of two new polyketides, namely epicoccofuran A (**1**) and flavimycin C (**2**). The latter was isolated as a diastereomeric mixture, along with four other known polyketides (**3**–**6**). Epicoccofuran A (**1**) and flavimycin C (**2**) displayed moderate activity against *B. subtilis* while flavimycin C (**2**), epicocconigrone A (**3**), epicoccone (**5**), and 4,5,6-trihydroxy-7-methyl-1,3-dihydroisobenzofuran (**6**) showed promising anticancer activities against seven tested cancer cell lines with the breast cancer cells being the most susceptible suggesting the use of these compounds (or their derivatives) as promising molecules for the development of anticancer therapeutics.

## Supplementary Information

Supplementary Figures.

## Data Availability

Data are available upon request from the first author, Mohamed S. Elnaggar.
