# Supplementary material for: Cytotoxic naphtho- and benzofurans from an endophytic fungus Epicoccum nigrum Ann-B-2 associated with Annona squamosa fruits
Source: Sci Rep. 2024 Feb 28;14:4940. doi: 10.1038/s41598-024-55168-5 (PMC10901772; doi:10.1038/s41598-024-55168-5)
Supplement: Supplementary file 1 — Supplementary Figures. [file 41598_2024_55168_MOESM1_ESM.pdf]

Supporting Information for:

Cytotoxic Naphtho- and Benzofurans from an Endophytic Fungus *Epicoccum nigrum* Ann-B-2 Associated with *Annona squamosa* Fruits

Mohamed S. Elnaggar[§,*], Shaimaa Fayez[§,*], Alaa Anwar[§], and Sherif S. Ebada[§,*]

[§] Department of Pharmacognosy, Faculty of Pharmacy, Ain Shams University, 11566 Cairo, Egypt

*** Corresponding Author**

E-mail: mohamed.s.elnaggar@pharma.asu.edu.eg (M.S.E.), Tel.: +20-2405-1180, Fax: +20-2405-1107; shaimaa_fayez@pharma.asu.edu.eg (S.F.), Tel.: +20-2405-1180, Fax: +20-2405-1107; sherif.elsayed@helmholtz-hzi.de; sherif_elsayed@pharma.asu.edu.eg (S.S.E.); Tel.: +49-531-6181-4223, Fax: +49-531-6181-9499

Table of Content

#		Page
**1**	Figure S1. HPLC chromatogram, UV and LR-ESI-MS spectra of **1**.	**S3**
**2**	Figure S2. HPLC chromatogram, UV and HR-ESI-MS spectra of **1**.	**S4**
**3**	Figure S3. ^{1}H NMR spectrum of **1** in DMSO-d_6 at 500 MHz.	**S5**
**4**	Figure S4. ^{13}C NMR spectrum of **1** in DMSO-d_6 at 125 MHz.	**S6**
**5**	Figure S5. HMBC spectrum of **1** in DMSO-d_6 at 500 MHz.	**S7**
**6**	Figure S6. HSQC spectrum of **1** in DMSO-d_6 at 500 MHz.	**S8**
**7**	Figure S7. ROESY spectrum of **1** in DMSO-d_6 at 500 MHz.	**S9**
**8**	Figure S8. HPLC chromatogram, UV and LR-ESI-MS spectra of **2**.	**S10**
**9**	Figure S9. HPLC chromatogram, UV and HR-ESI-MS spectra of **2**.	**S11-S12**
**10**	Figure S10. ^{1}H NMR spectrum of **2** in DMSO-d_6 at 700 MHz.	**S13**
**11**	Figure S11. ^{13}C NMR spectrum of **2** in DMSO-d_6 at 175 MHz.	**S14**
**12**	Figure S12. ^{1}H-^{1}H COSY spectrum of **2** in DMSO-d_6 at 700 MHz.	**S15**
**13**	Figure S13. HMBC spectrum of **2** in DMSO-d_6 at 700 MHz.	**S16**
**14**	Figure S14. HSQC spectrum of **2** in DMSO-d_6 at 700 MHz.	**S17**
**15**	Figure S15. ROESY spectrum of **2** in DMSO-d_6 at 700 MHz.	**S18**
**16**	Figure S16. HPLC chromatogram, UV and LR-ESI-MS spectra of **3**.	**S19**
**17**	Figure S17. ^{1}H NMR spectrum of **3** in DMSO-d_6 at 500 MHz.	**S20**
**18**	Figure S18. ^{13}C NMR spectrum of **3** in DMSO-d_6 at 125 MHz.	**S21**
**19**	Figure S19. HMBC spectrum of **3** in DMSO-d_6 at 500 MHz.	**S22**
**20**	Figure S20. ROESY spectrum of **3** in DMSO-d_6 at 500 MHz.	**S23**
**21**	Figure S21. HPLC chromatogram, UV and LR-ESI-MS spectra of **4**.	**S24**
**22**	Figure S22. ^{1}H NMR spectrum of **4** in DMSO-d_6 at 500 MHz.	**S25**
**23**	Figure S23. HMBC spectrum of **4** in DMSO-d_6 at 500 MHz.	**S26**
**24**	Figure S24. HSQC spectrum of **4** in DMSO-d_6 at 500 MHz.	**S27**
**25**	Figure S25. ROESY spectrum of **4** in DMSO-d_6 at 500 MHz.	**S28**
**26**	Figure S26. HPLC chromatogram, UV and LR-ESI-MS spectra of **5**.	**S29**
**27**	Figure S27. HPLC chromatogram, UV and HR-ESI-MS spectra of **5**.	**S30**
**28**	Figure S28. ^{1}H NMR spectrum of **5** in DMSO-d_6 at 500 MHz.	**S31**
**29**	Figure S29. ^{13}C NMR spectrum of **5** in DMSO-d_6 at 125 MHz.	**S32**
**30**	Figure S30. HMBC spectrum of **5** in DMSO-d_6 at 500 MHz.	**S33**
**31**	Figure S31. HSQC spectrum of **5** in DMSO-d_6 at 500 MHz.	**S34**
**32**	Figure S32. ROESY spectrum of **5** in DMSO-d_6 at 500 MHz.	**S35**
**33**	Figure S33. HPLC chromatogram, UV and LR-ESI-MS spectra of **6**.	**S36**
**34**	Figure S34. HPLC chromatogram, UV and HR-ESI-MS spectra of **6**.	**S37**
**35**	Figure S35. ^{1}H NMR spectrum of **6** in DMSO-d_6 at 500 MHz.	**S38**
**36**	Figure S36. ^{13}C NMR spectrum of **6** in DMSO-d_6 at 125 MHz.	**S39**
**37**	Figure S37. HMBC spectrum of **6** in DMSO-d_6 at 500 MHz.	**S40**
**38**	Figure S38. HSQC spectrum of **6** in DMSO-d_6 at 500 MHz.	**S41**
**39**	Figure S39. ROESY spectrum of **6** in DMSO-d_6 at 500 MHz.	**S42**

Generic Display Report

Analysis Info

Analysis Name	C:\SEL22\Amazon\ANN-B-2\A10.H20.5a.2_BB3_01_42549.d	Acquisition Date	23.10.2022 18:26:14
Method	42549.m	Operator	esu
Sample Name	A10.H20.5a.2	Instrument	amaZon speed
Comment			

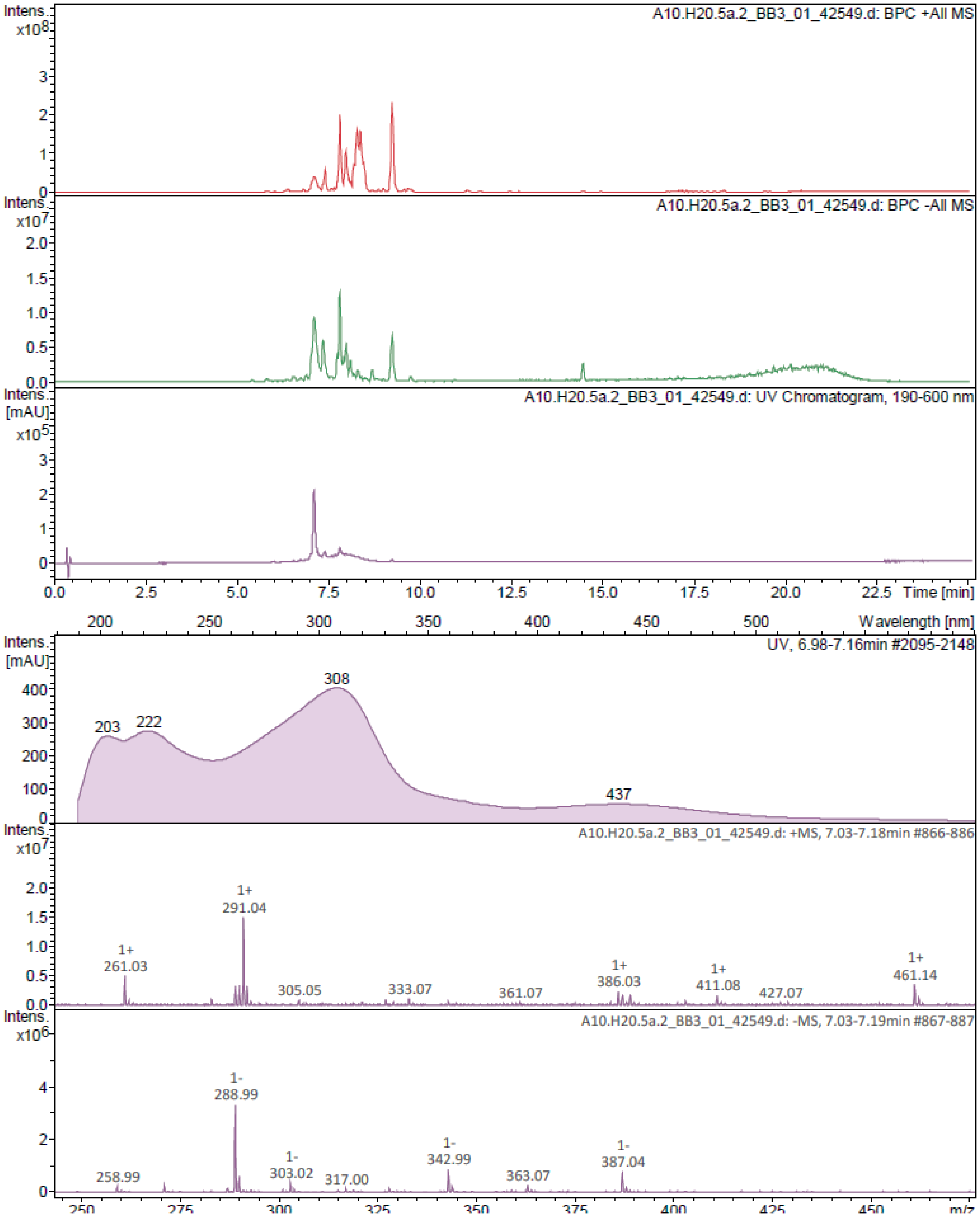

Bruker Compass DataAnalysis 4.4 printed: 02.11.2022 16:27:53 by: sel22 Page 1 of 1

Figure S1. HPLC chromatogram, UV and LR-ESI-MS spectra of **1**.

Generic Display Report

Analysis Info

		Acquisition Date	11.11.2022 14:32:27
Analysis Name	S:\DATA\MaXis\sel22_SherifElsayed\22_11\A10.H2O.5a.2_44_01_10996.d		
Method	pos_säure_10000_screening_ms_100_2500_line.m	Operator	ate06
Sample Name	A10.H2O.5a.2	Instrument	maXis
Comment	Screening01<br>Waters Acquity UPLC BEH C_18 1,7um 2.1x50mm		

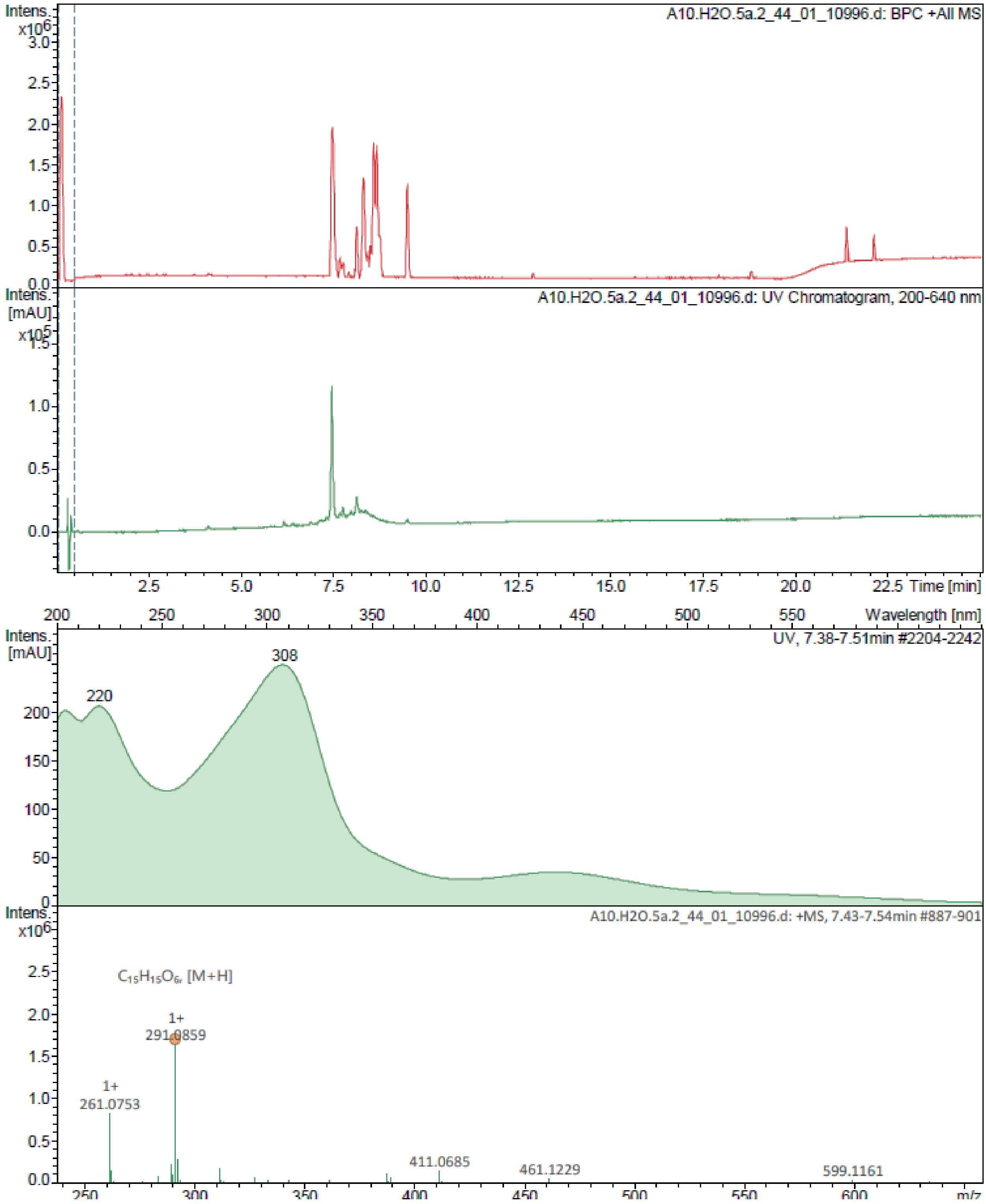

Bruker Compass DataAnalysis 4.4	printed: 14.11.2022 11:21:30	by: sel22	Page 1 of 1

Figure S2. HPLC chromatogram, UV and HR-ESI-MS spectra of **1**.

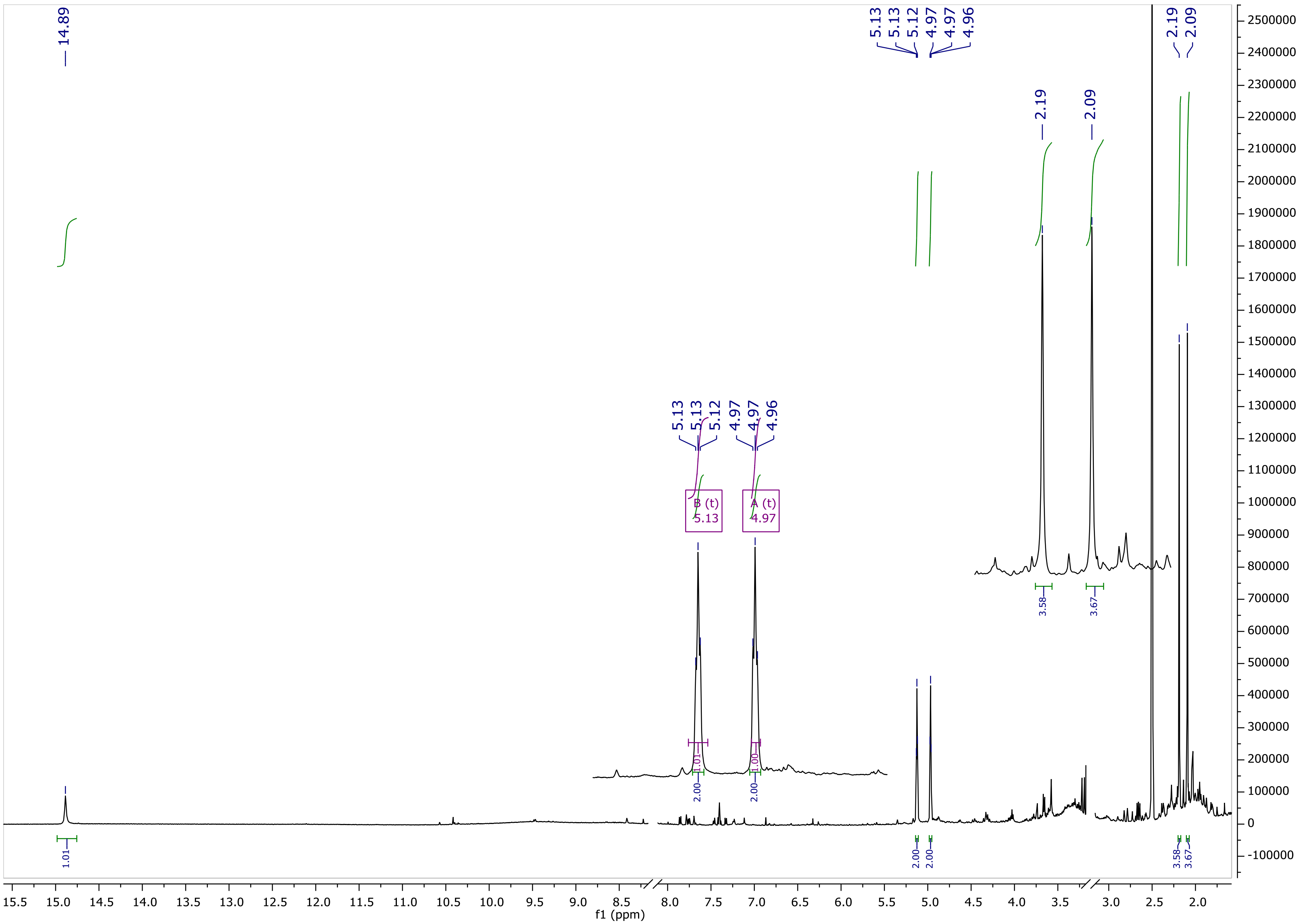

Figure S3. ^{1}H NMR spectrum of **1** in DMSO-d_6 at 500 MHz.

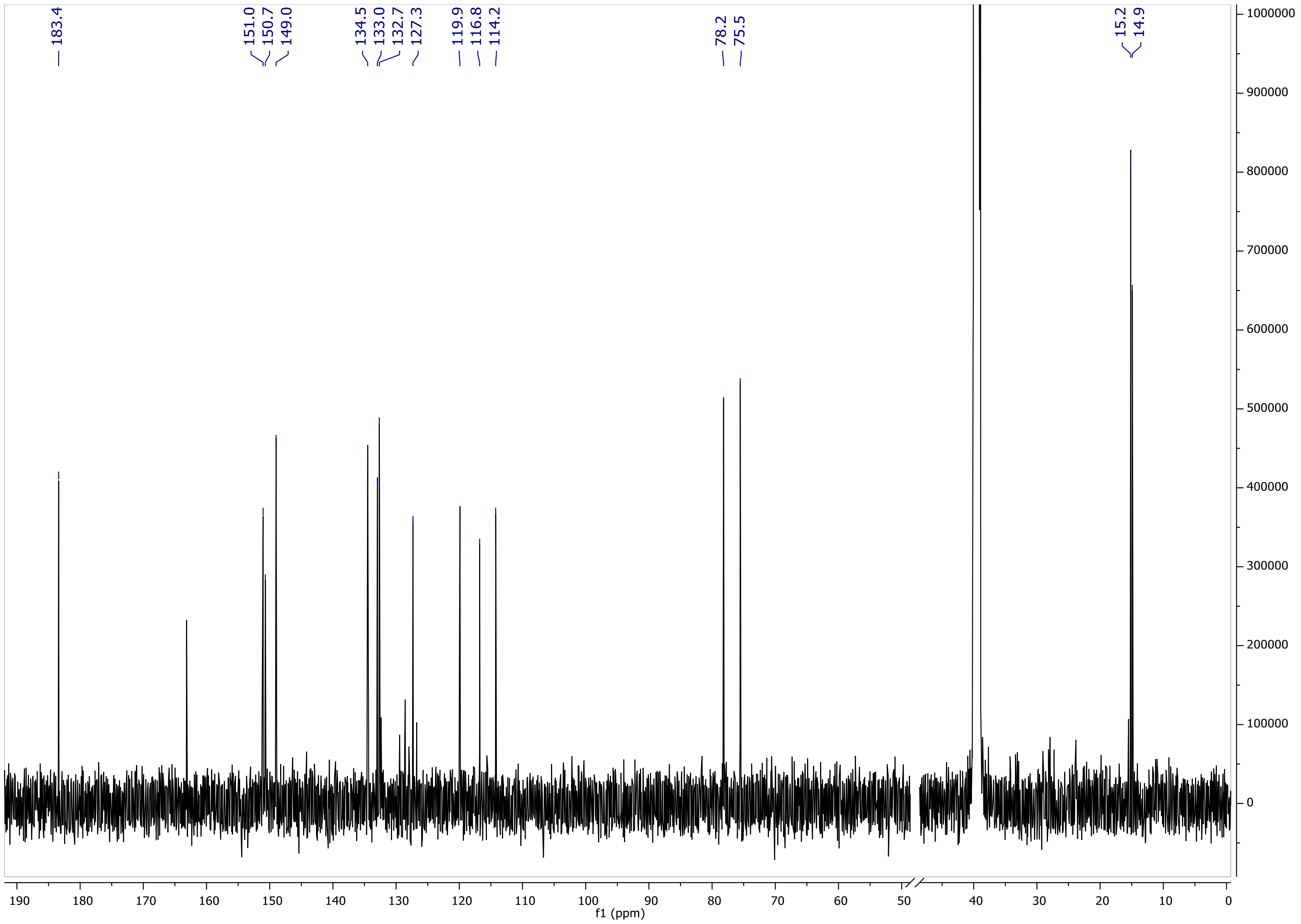

Figure S4. ^{13}C NMR spectrum of **1** in DMSO-d_6 at 125 MHz.

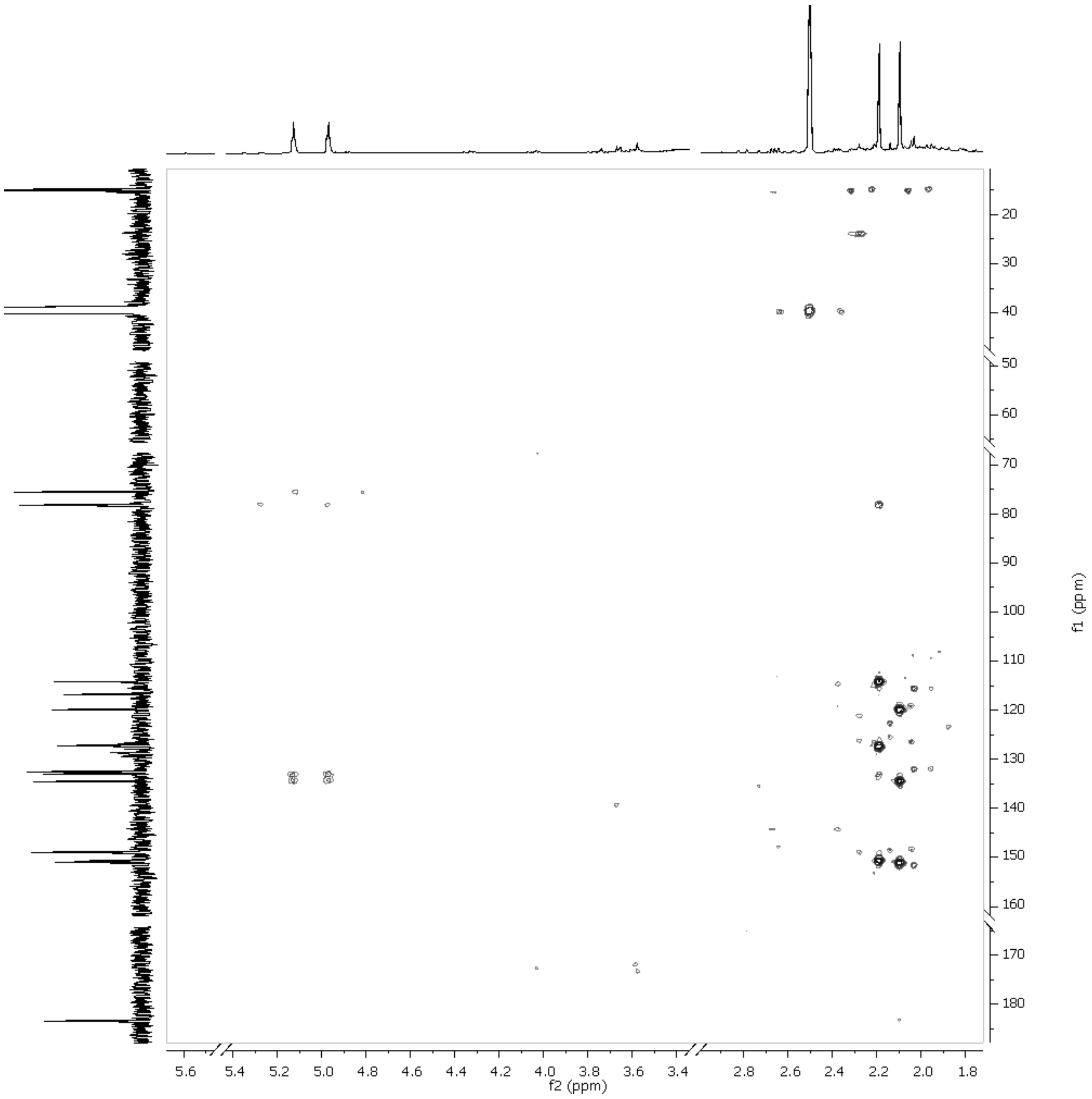

Figure S5. HMBC spectrum of **1** in DMSO-d_6 at 500 MHz.

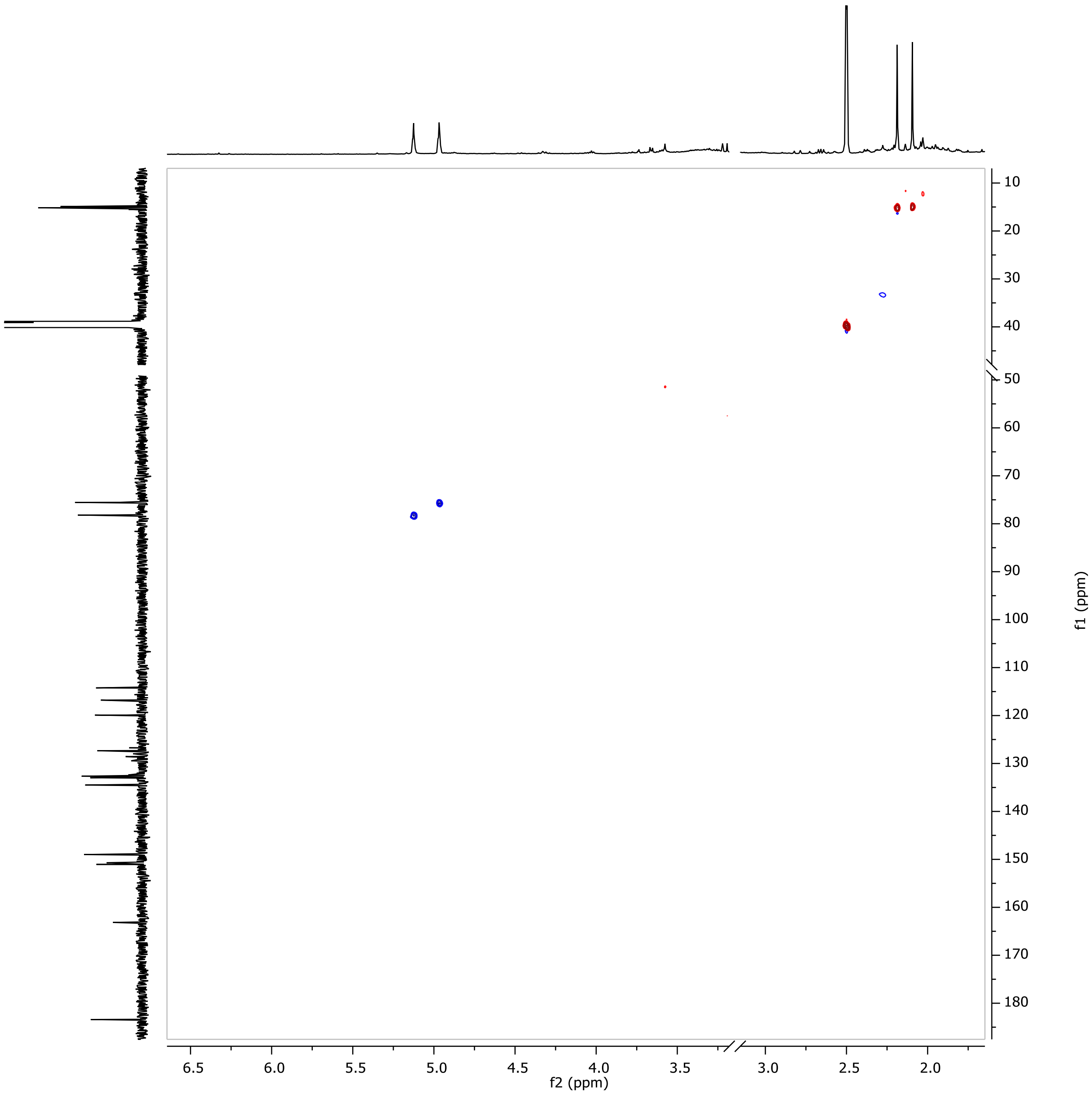

Figure S6. HSQC spectrum of **1** in DMSO-d_6 at 500 MHz.

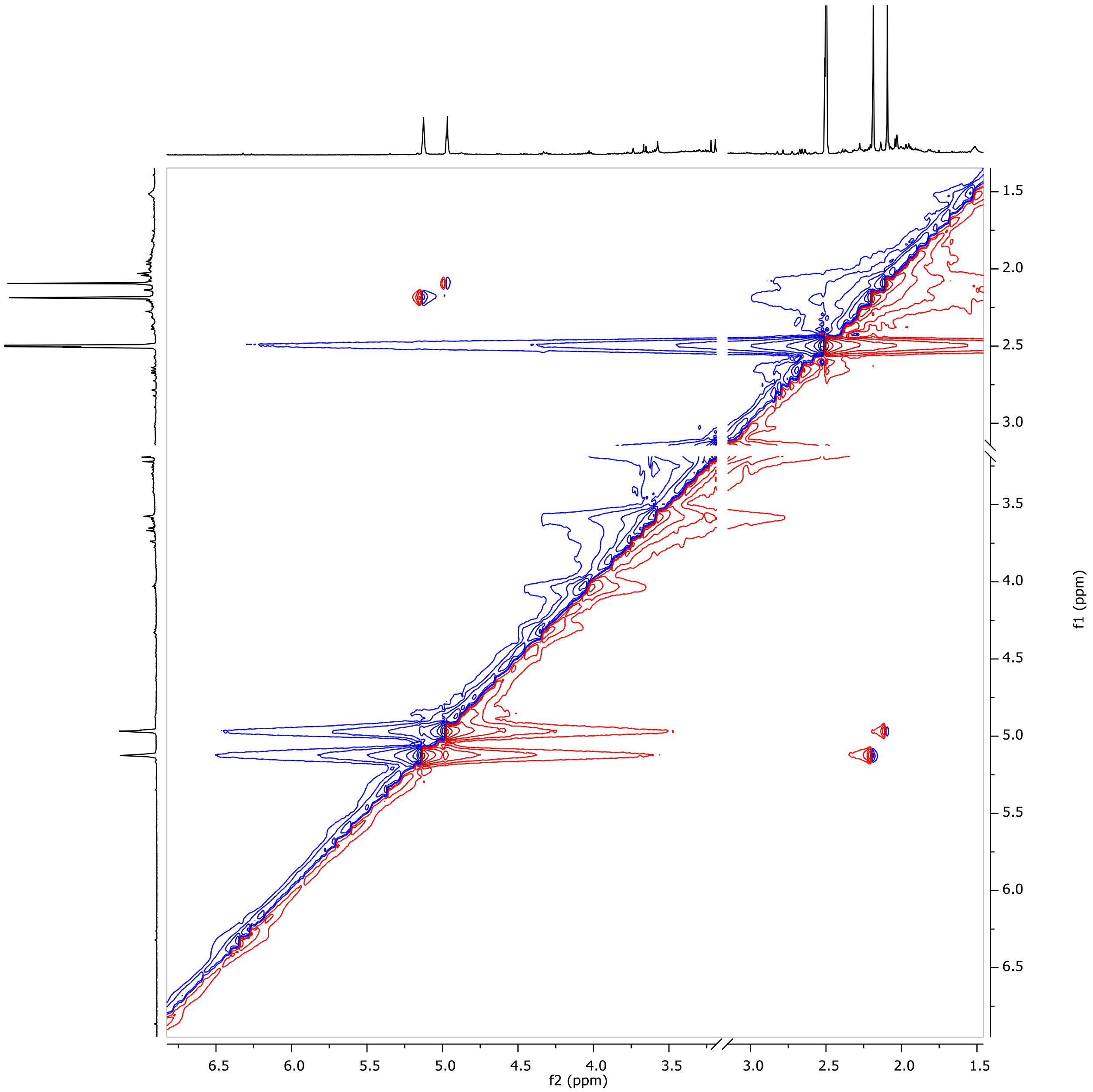

Figure S7. ROESY spectrum of **1** in DMSO-d_6 at 500 MHz.

Generic Display Report

Analysis Info

Analysis Name	C:\SEL22\Amazon\ANN-B-2\A10.H20.5.1_BA1_01_42536.d	Acquisition Date	22.10.2022 22:42:55
Method	42536.m	Operator	esu
Sample Name	A10.H20.5.1	Instrument	amaZon speed
Comment			

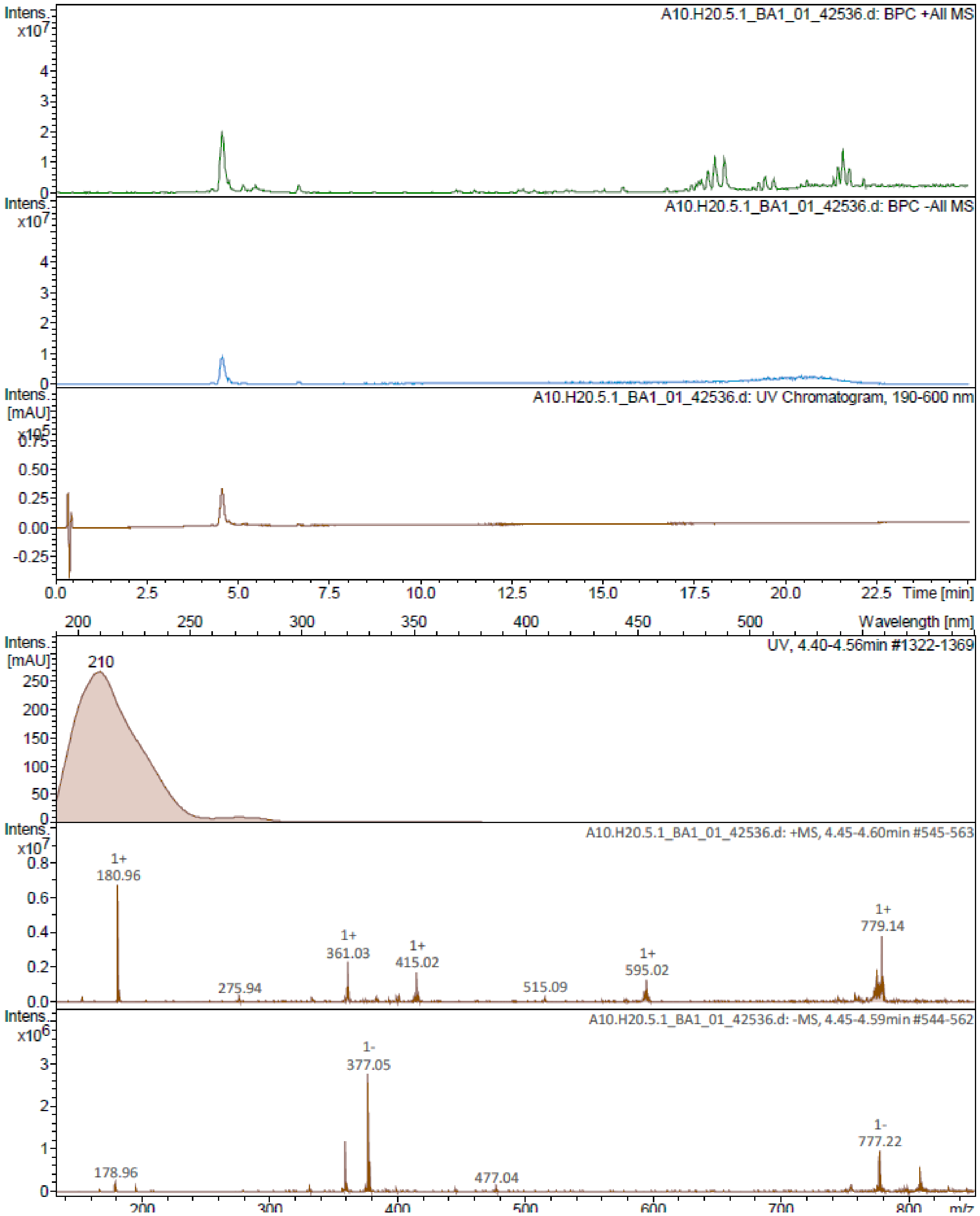

Bruker Compass DataAnalysis 4.4 printed: 02.11.2022 16:30:23 by: sel22 Page 1 of 1

Figure S8. HPLC chromatogram, UV and LR-ESI-MS spectra of **2**.

Generic Display Report

Analysis Info | Acquisition Date 11.11.2022 13:30:30

Analysis Name	S:\DATA\MaXis\sel22_Sherif Elsayed\22_11_MAxis\A10.H2O.5.1_42_01_10994.d		
Method	pos_säure_10000_screening_ms_100_2500_line.m	Operator	ate06
Sample Name	A10.H2O.5.1	Instrument	maXis
Comment	Screening01		
	Waters Acquity UPLC BEH C_18 1,7um 2.1x50mm		

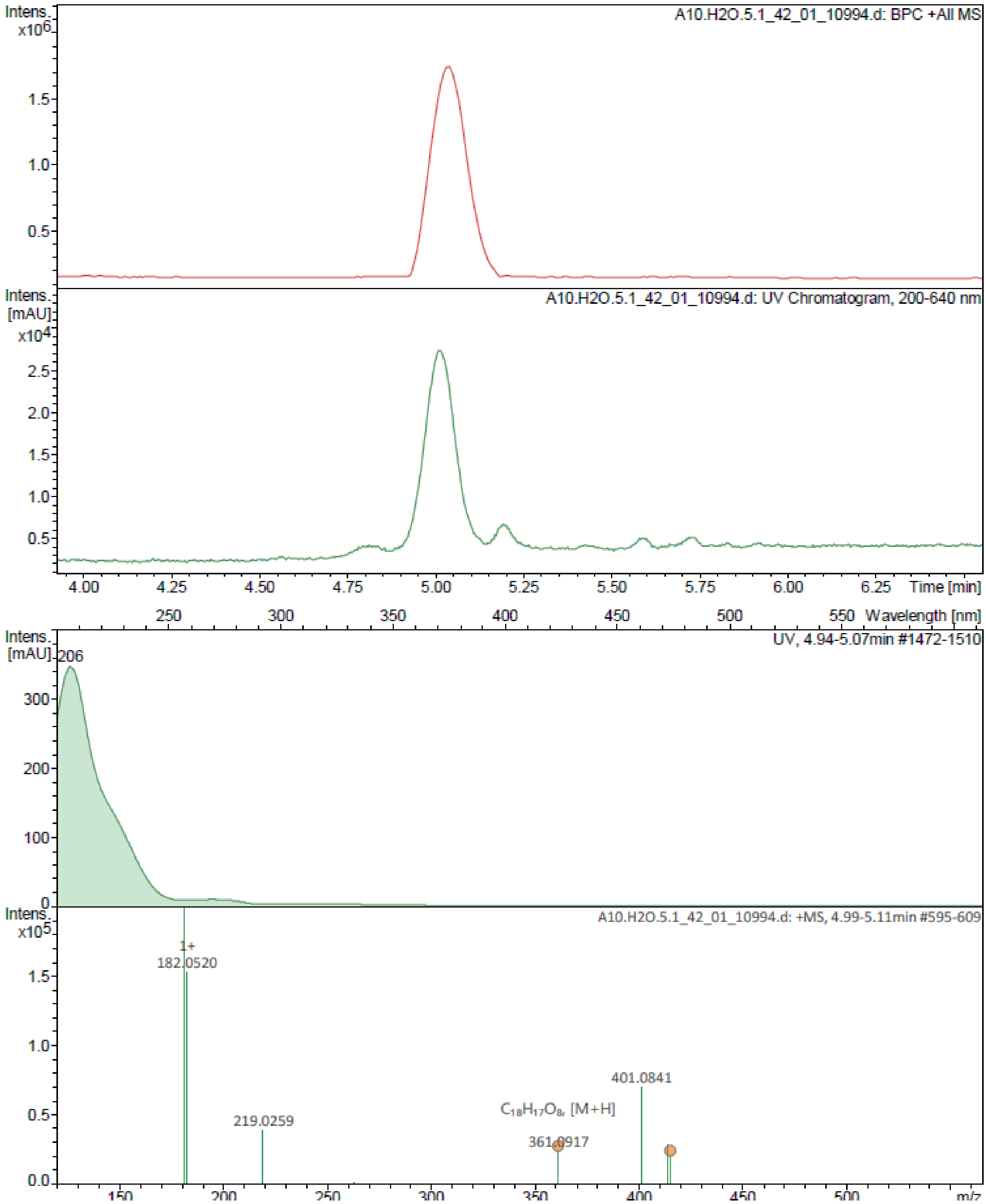

Generic Display Report

Analysis Info

Analysis Name	S:\DATA\MaXis\sel22_SherifElsayed\22_11_MAxis\A10.H20.5.1_neg_11_01_11031.d	Acquisition Date	21.11.2022 11:39:27
Method	neg_säure_10000_screening_ms_100_2500_line+profile.m	Operator	ate06
Sample Name	A10.H20.5.1_neg	Instrument	maXis
Comment	Screening01<br>Waters Acquity UPLC BEH C_18 1,7um 2.1x50mm		

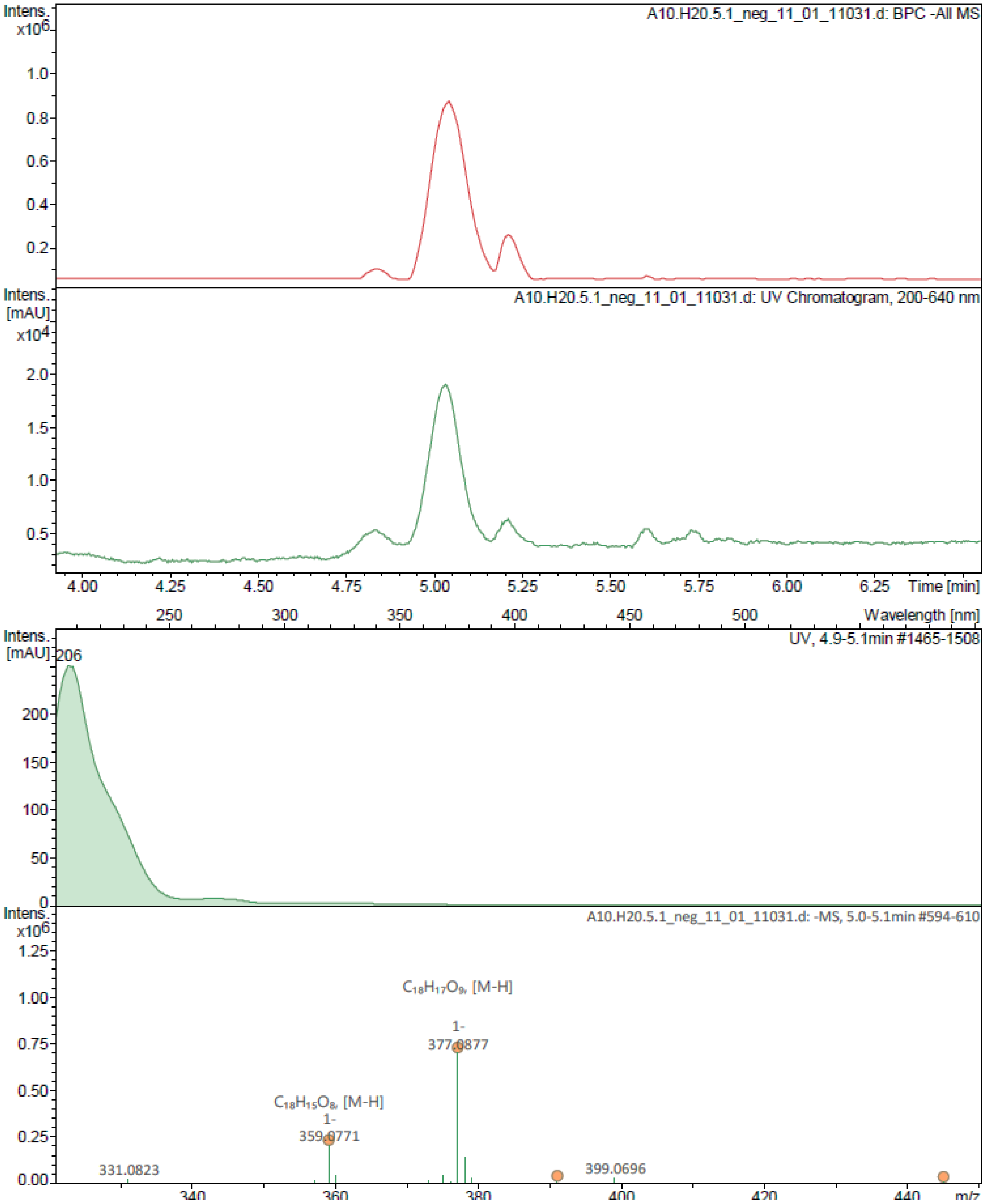

Bruker Compass DataAnalysis 4.4 printed: 05.12.2022 14:36:10 by: sel22 Page 1 of 1

Figure S9. HPLC chromatogram, UV and HR-ESI-MS spectra of **2**.

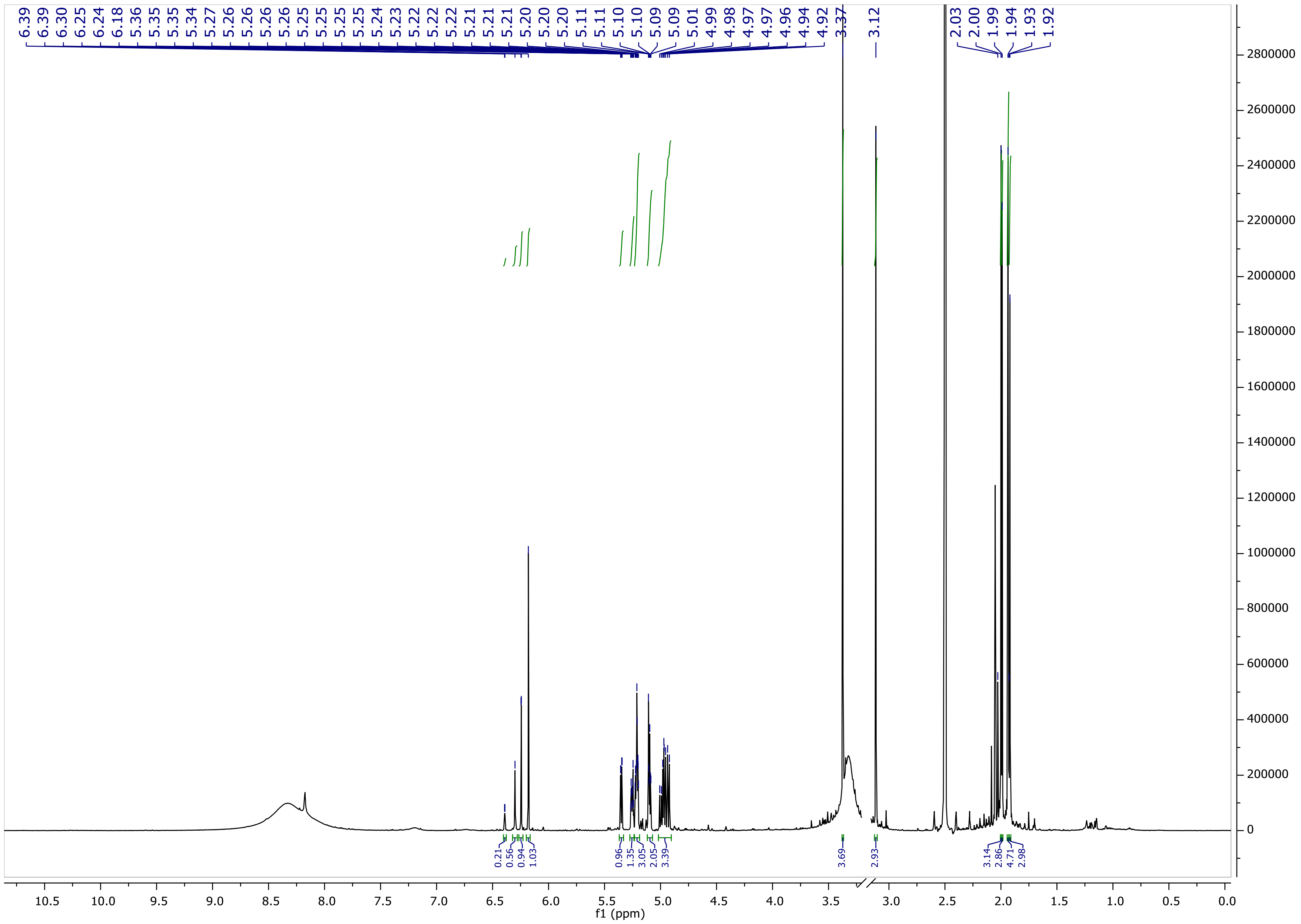

Figure S10. ^{1}H NMR spectrum of **2** in DMSO-d_6 at 700 MHz.

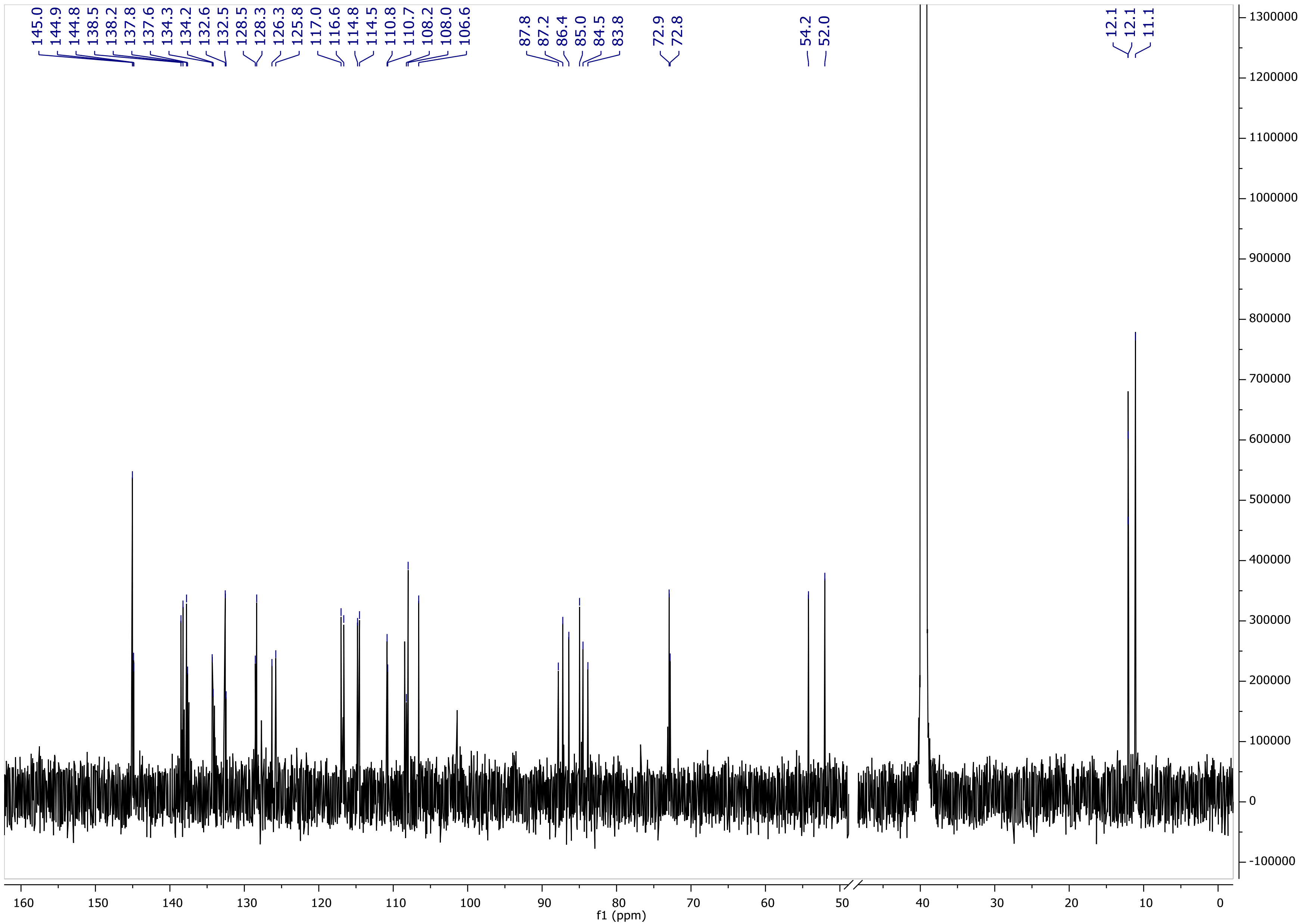

Figure S11. ^{13}C NMR spectrum of **2** in DMSO-d_6 at 175 MHz.

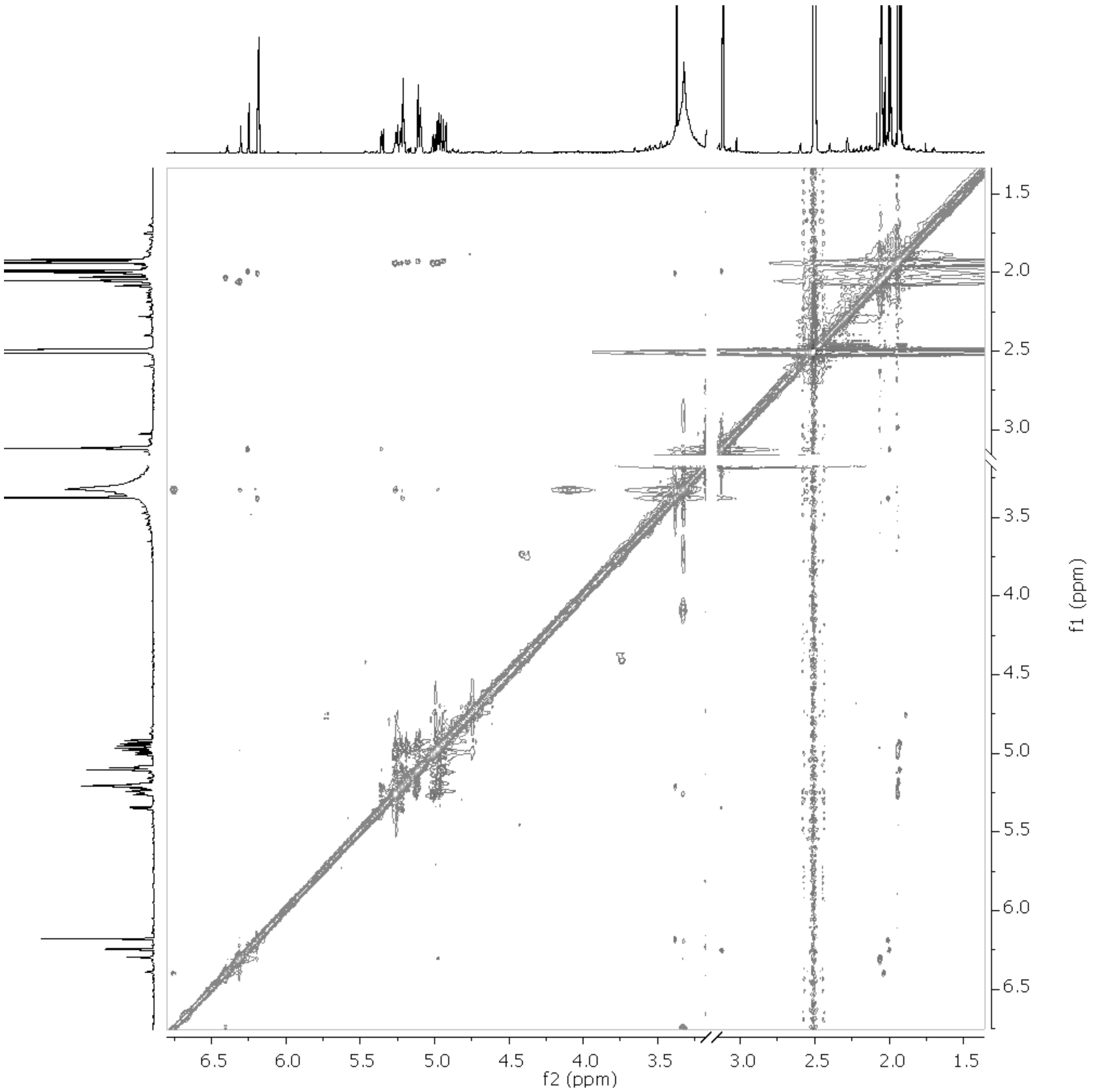

Figure S12. ^{1}H-^{1}H COSY spectrum of **2** in DMSO-d_6 at 700 MHz.

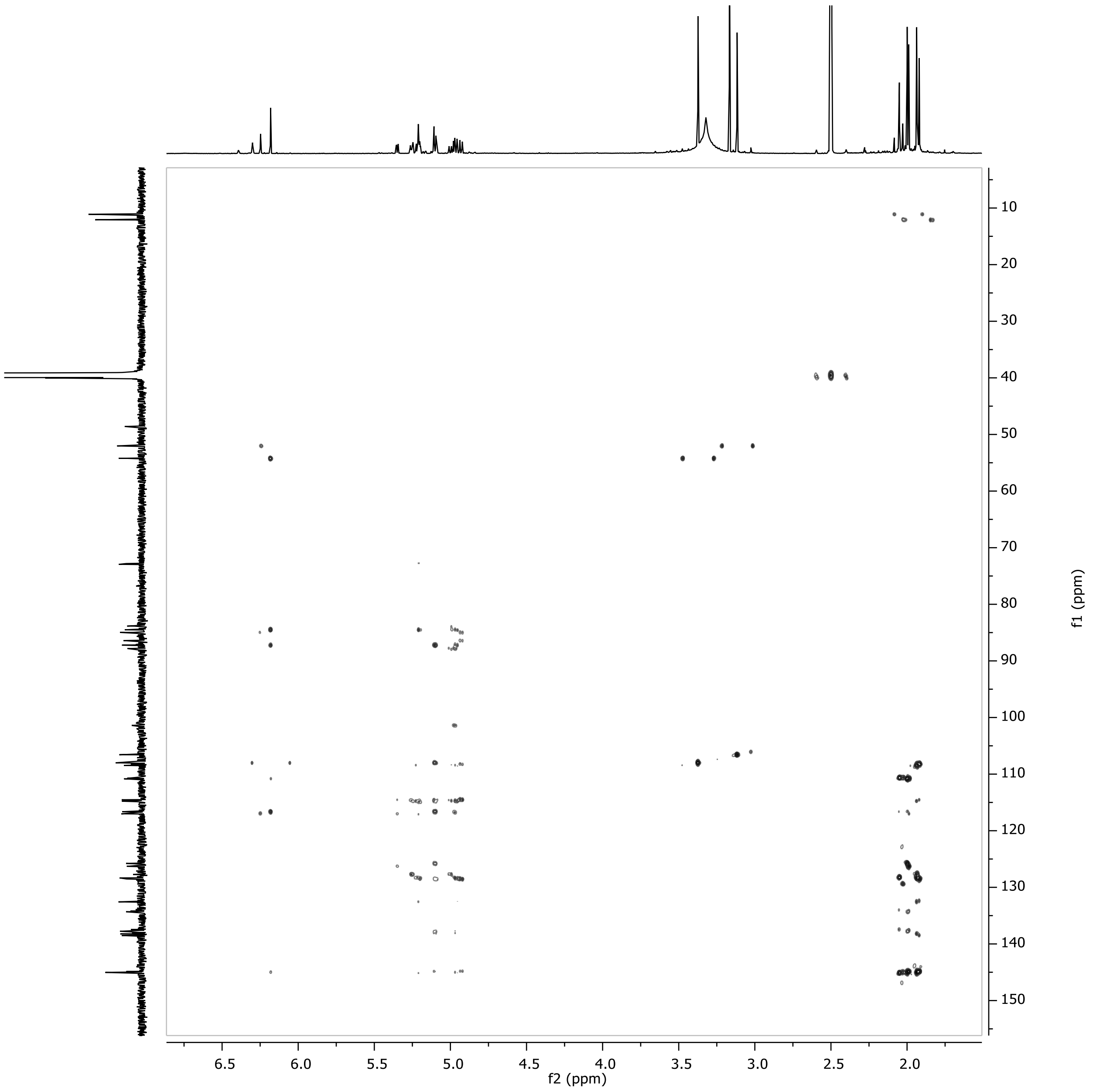

Figure S13. HMBC spectrum of **2** in DMSO-d_6 at 700 MHz.

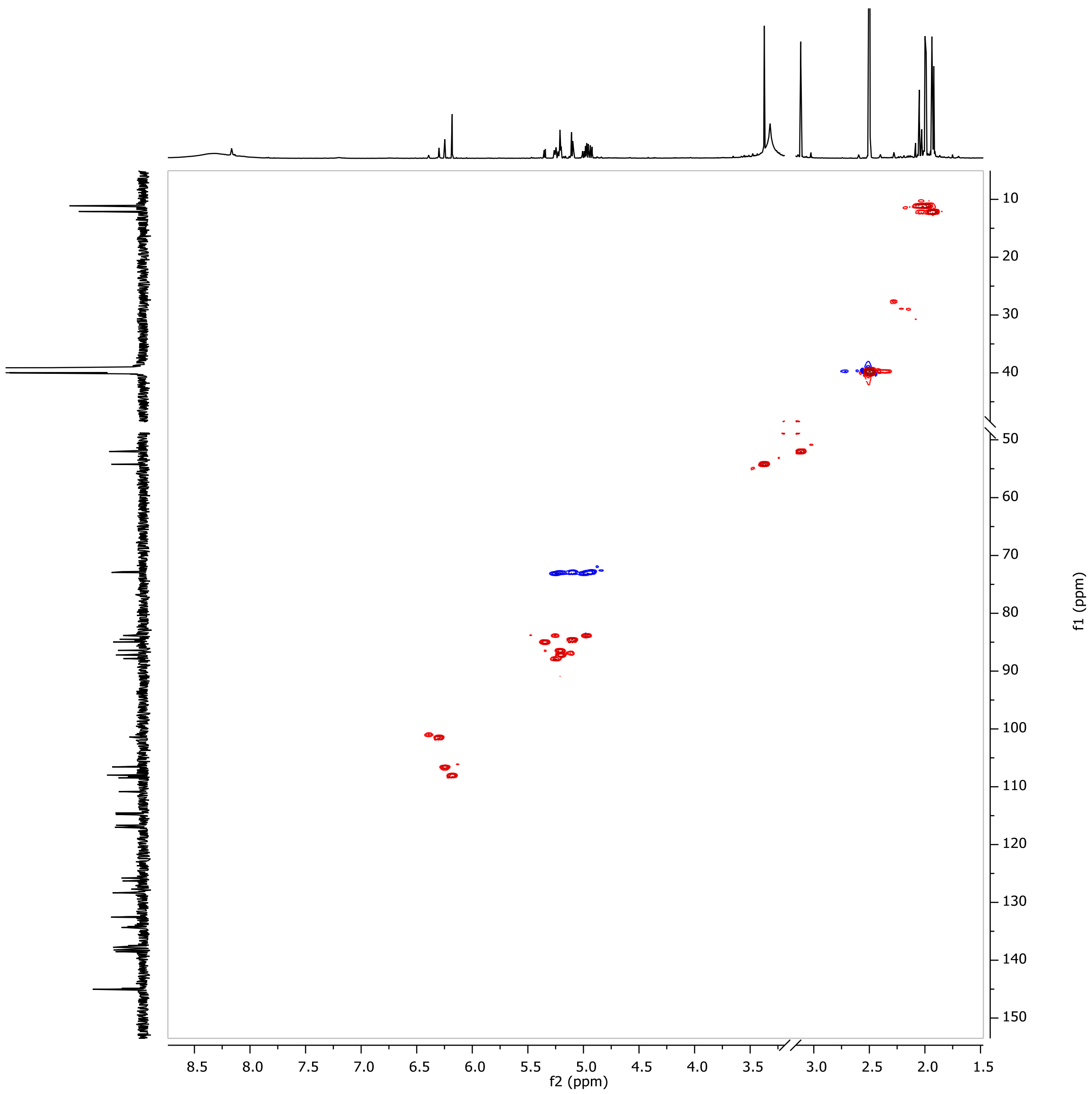

Figure S14. HSQC spectrum of **2** in DMSO-d_6 at 700 MHz.

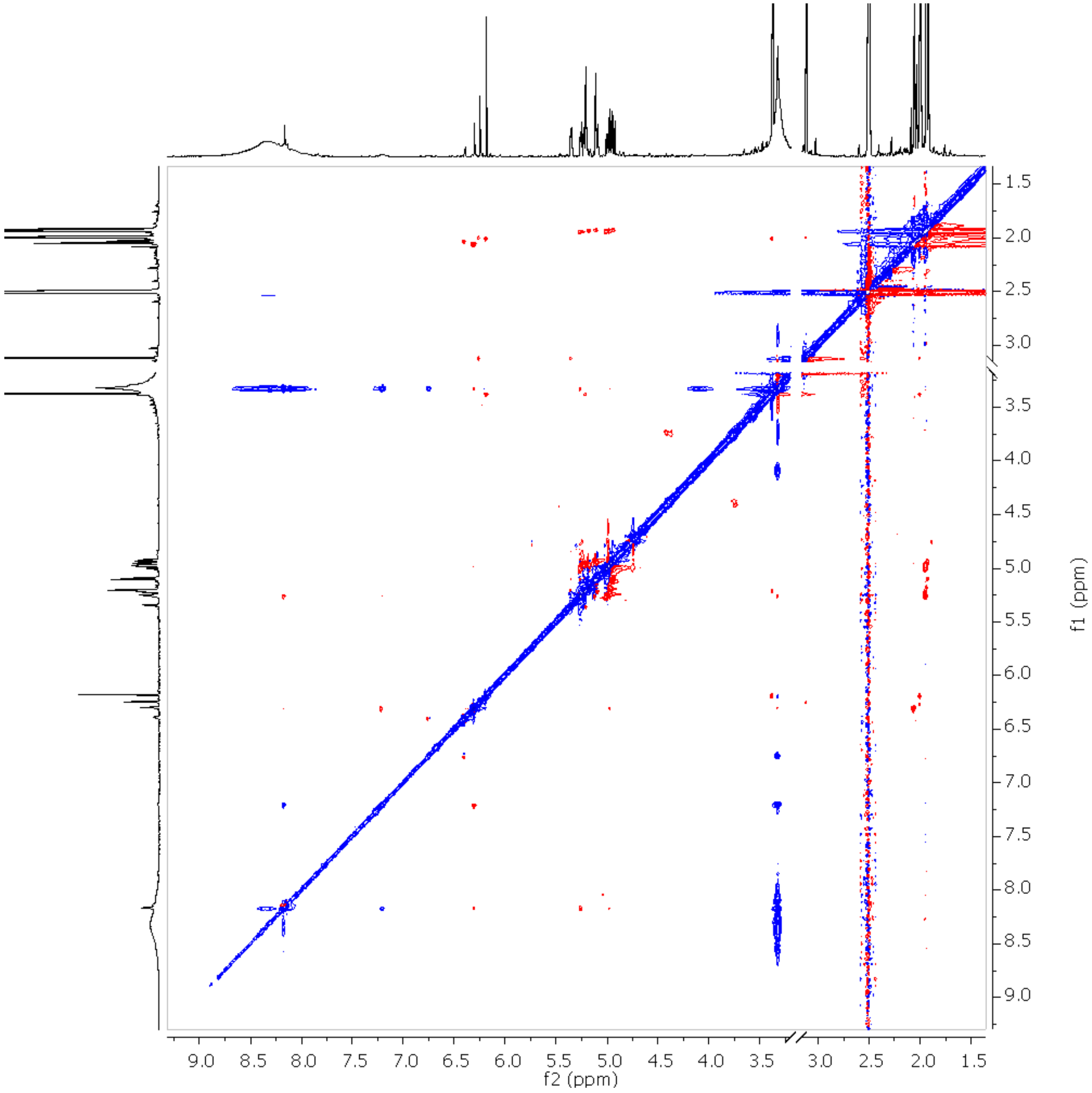

Figure S15. ROESY spectrum of **2** in DMSO-d_6 at 700 MHz.

Generic Display Report

Analysis Info

Analysis Name	C:\SEL22\Amazon\ANN-B-2\A10.H20.5a.1_BB2_01_42548.d	Acquisition Date	23.10.2022 17:50:00
Method	42548.m	Operator	esu
Sample Name	A10.H20.5a.1	Instrument	amaZon speed
Comment			

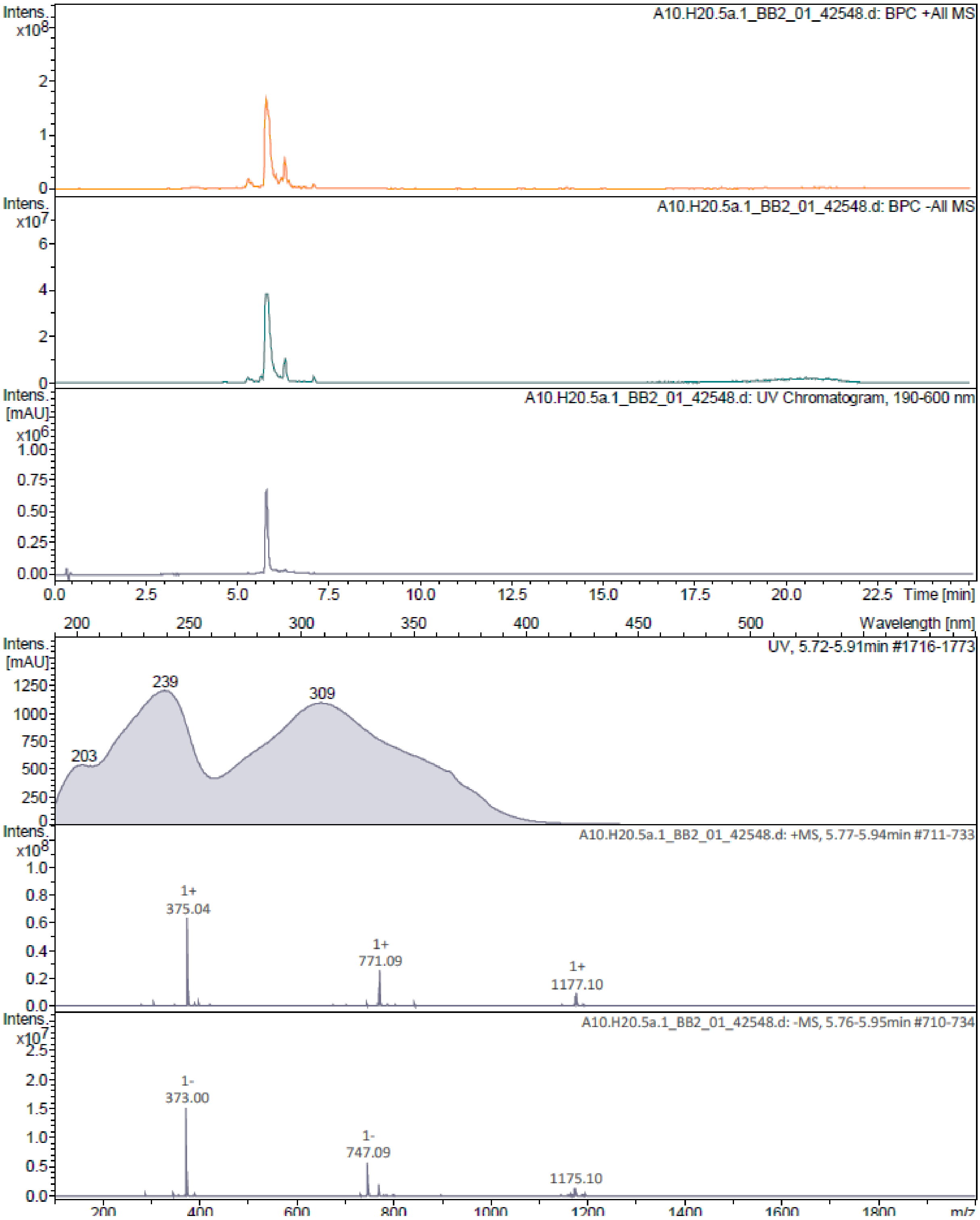

Bruker Compass DataAnalysis 4.4 printed: 02.11.2022 16:28:48 by: sel22 Page 1 of 1

Figure S16. HPLC chromatogram, UV and LR-ESI-MS spectra of **3**.

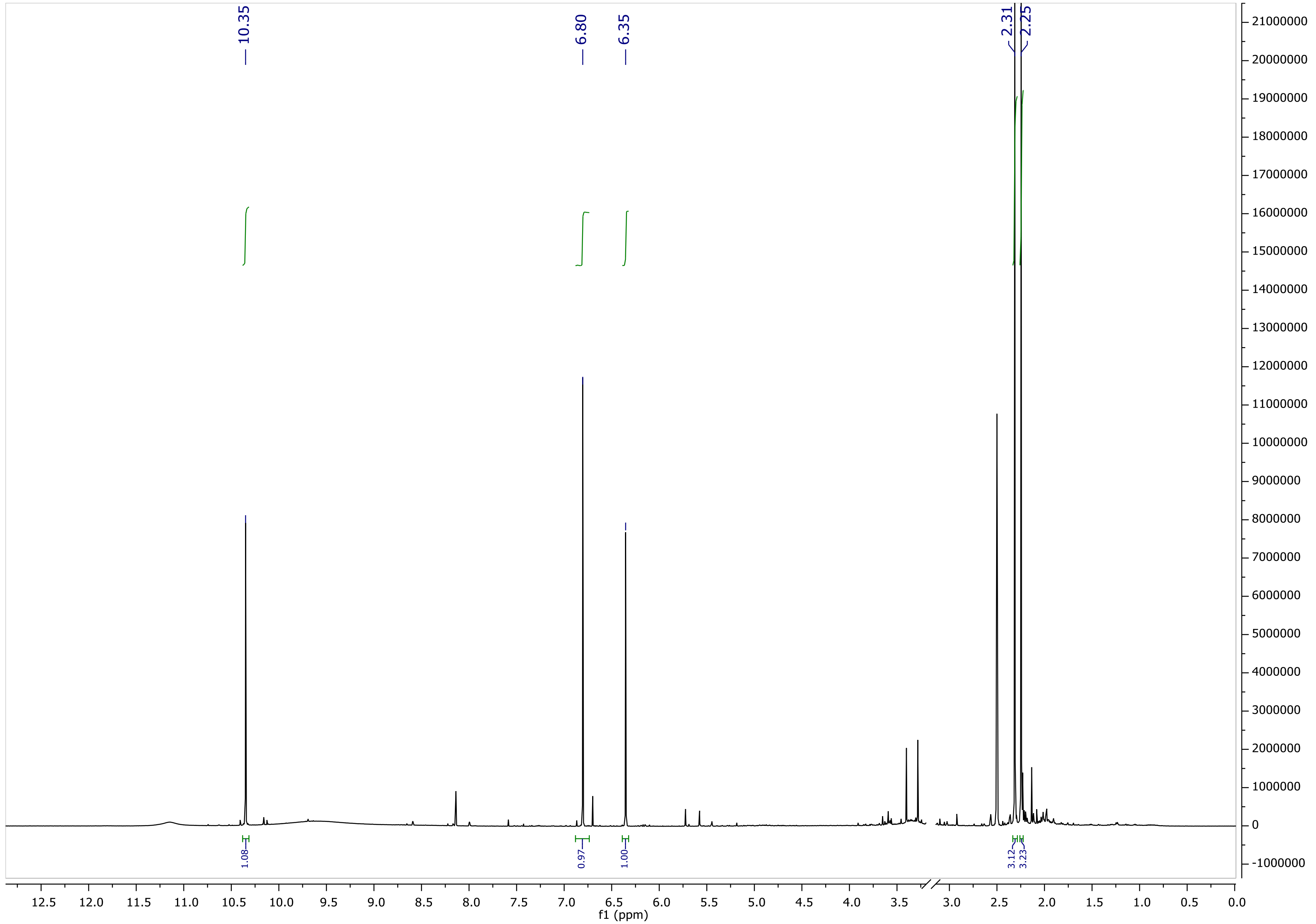

Figure S17. ^{1}H NMR spectrum of **3** in DMSO-d_6 at 500 MHz.

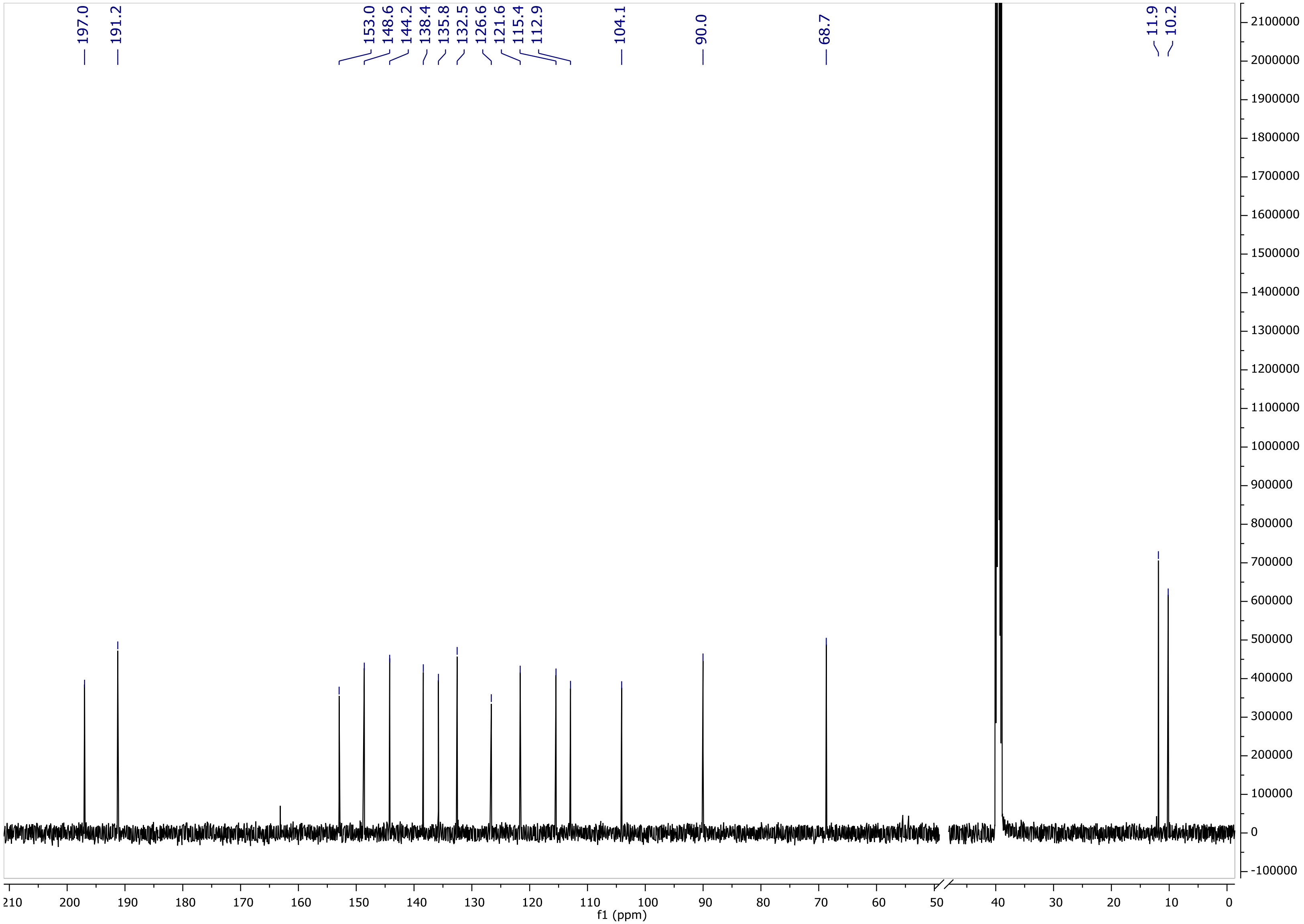

Figure S18. ^{13}C NMR spectrum of **3** in DMSO-d_6 at 125 MHz.

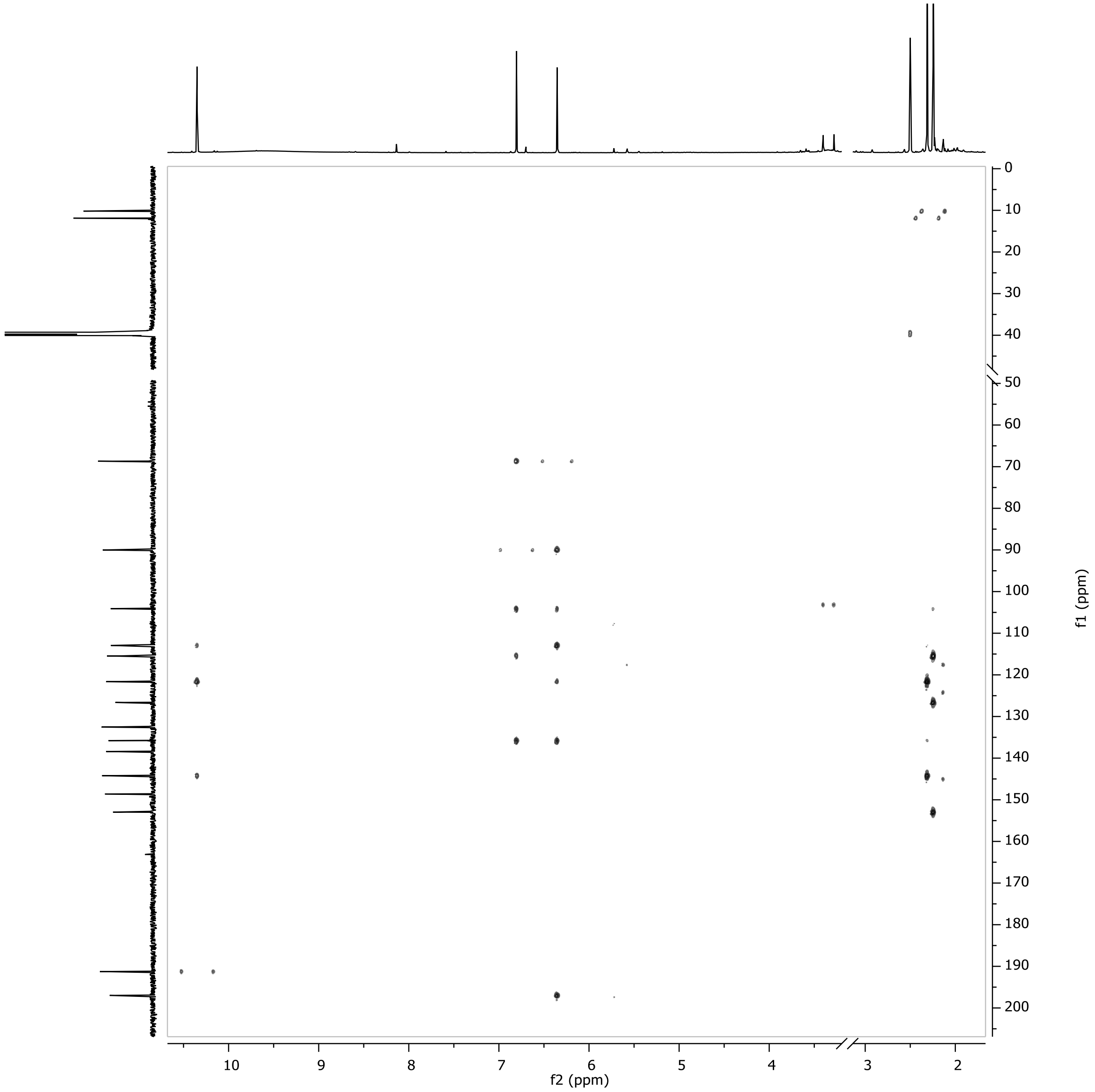

Figure S19. HMBC spectrum of **3** in DMSO-d_6 at 500 MHz.

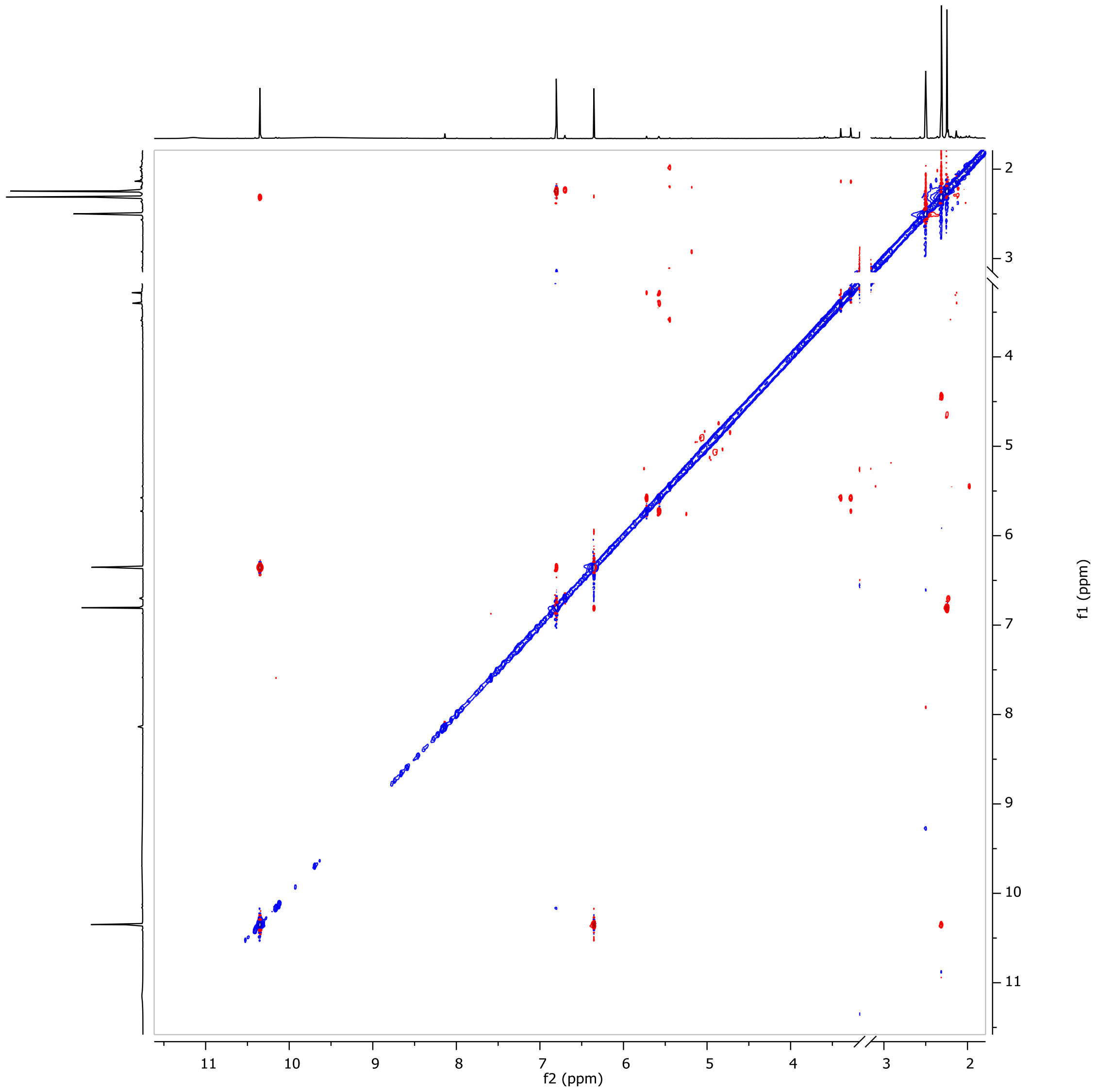

Figure S20. ROESY spectrum of **3** in DMSO-d_6 at 500 MHz.

Generic Display Report

Analysis Info

Analysis Name	C:\SEL22\Amazon\ANN-B-2\A10.H20.5.5_BA5_01_42540.d	Acquisition Date	23.10.2022 01:07:35
Method	42540.m	Operator	esu
Sample Name	A10.H20.5.5	Instrument	amaZon speed
Comment			

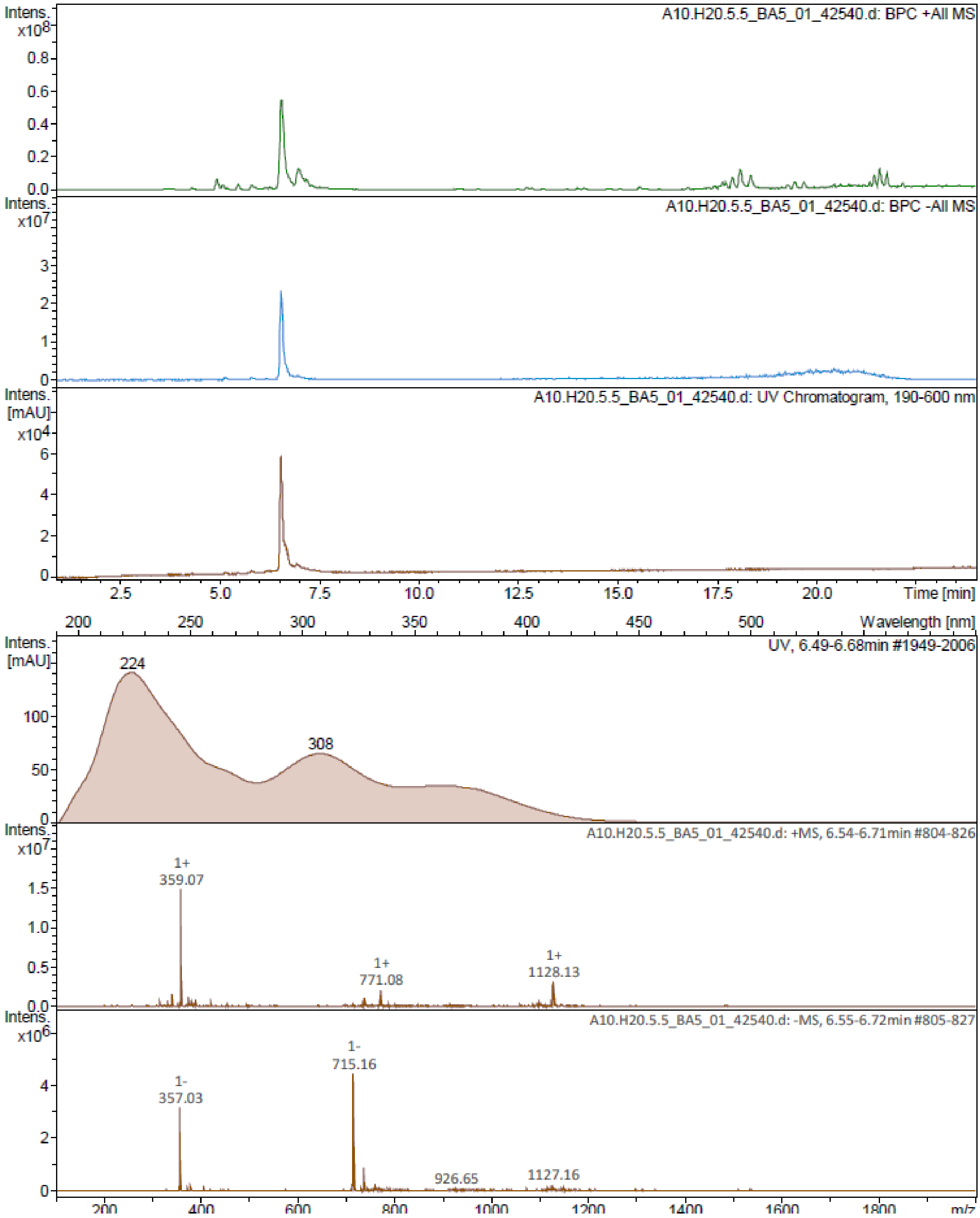

Bruker Compass DataAnalysis 4.4 printed: 07.11.2022 11:58:12 by: sel22 Page 1 of 1

Figure S21. HPLC chromatogram, UV and LR-ESI-MS spectra of **4**.

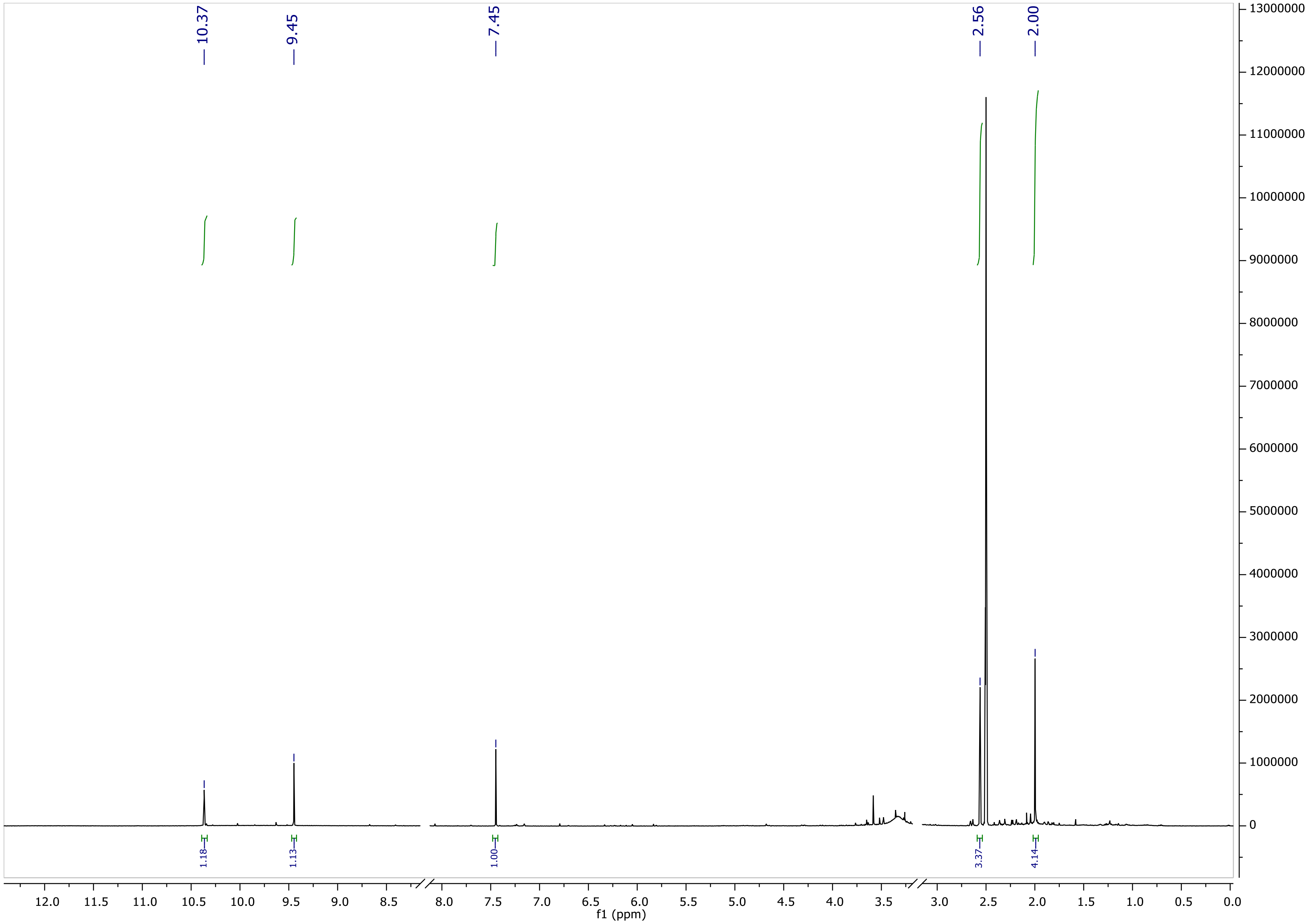

Figure S22. ^{1}H NMR spectrum of **4** in DMSO-d_6 at 500 MHz.

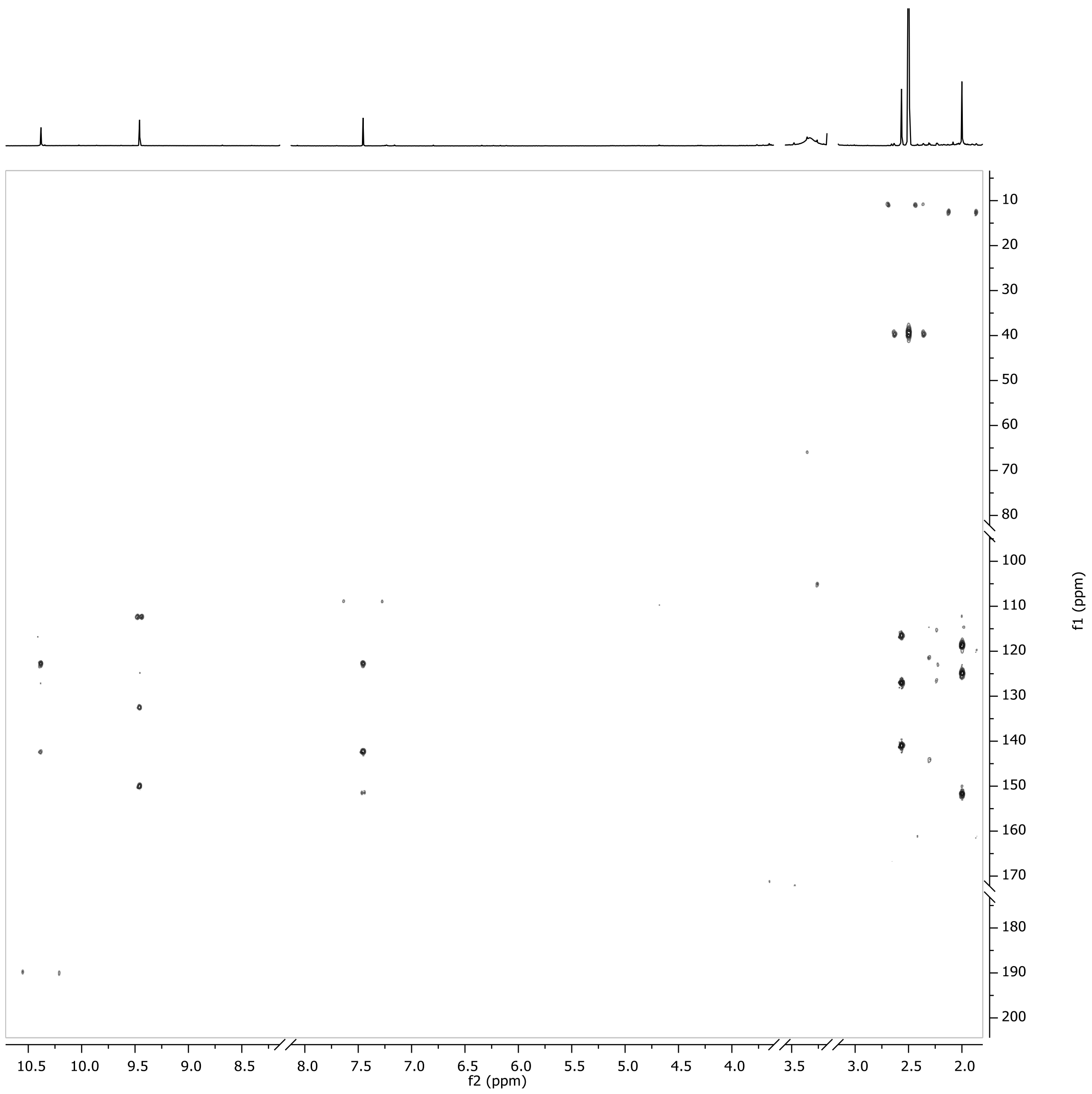

Figure S23. HMBC spectrum of **4** in DMSO-d_6 at 500 MHz.

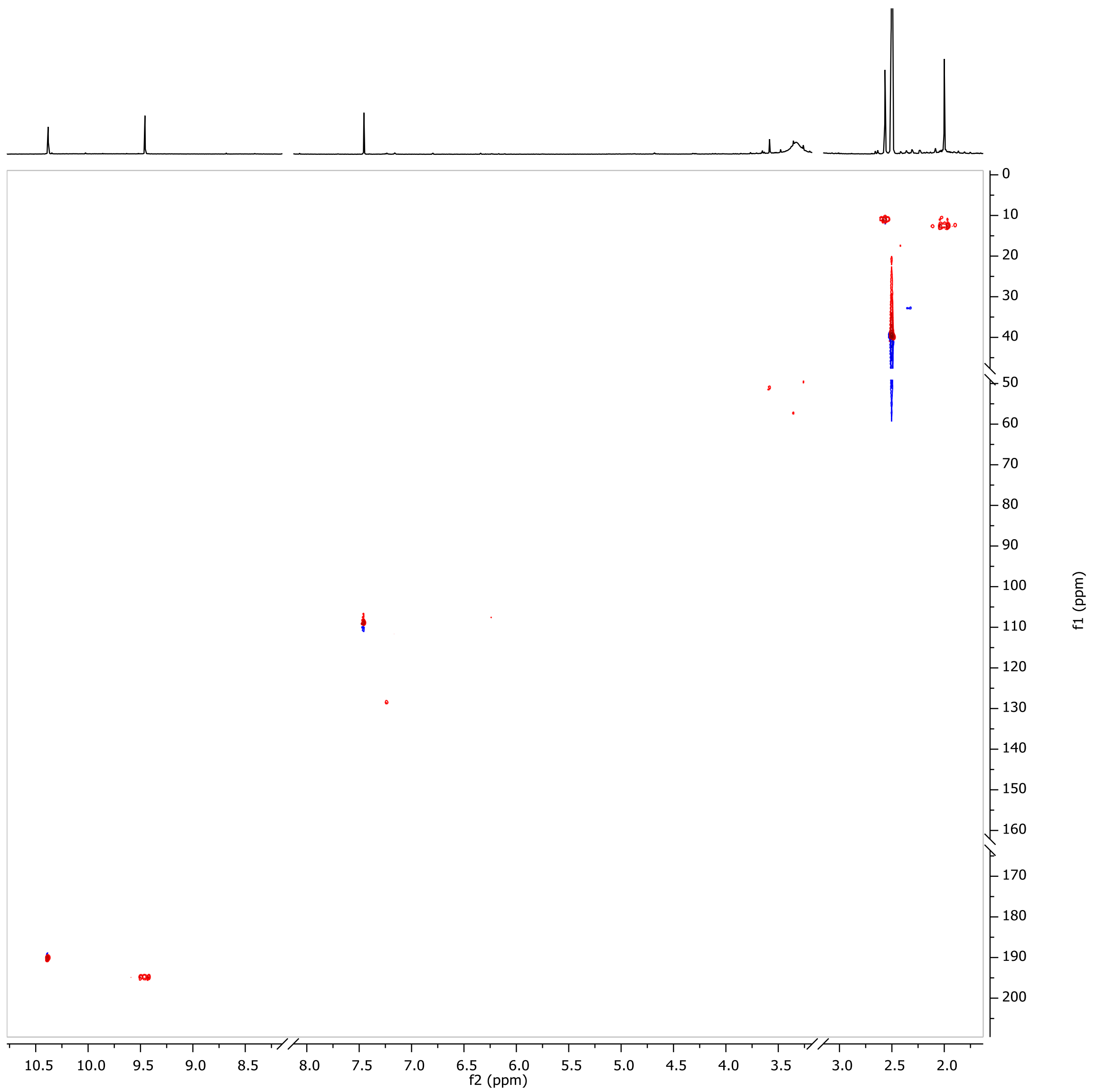

Figure S24. HSQC spectrum of **4** in DMSO-d_6 at 500 MHz.

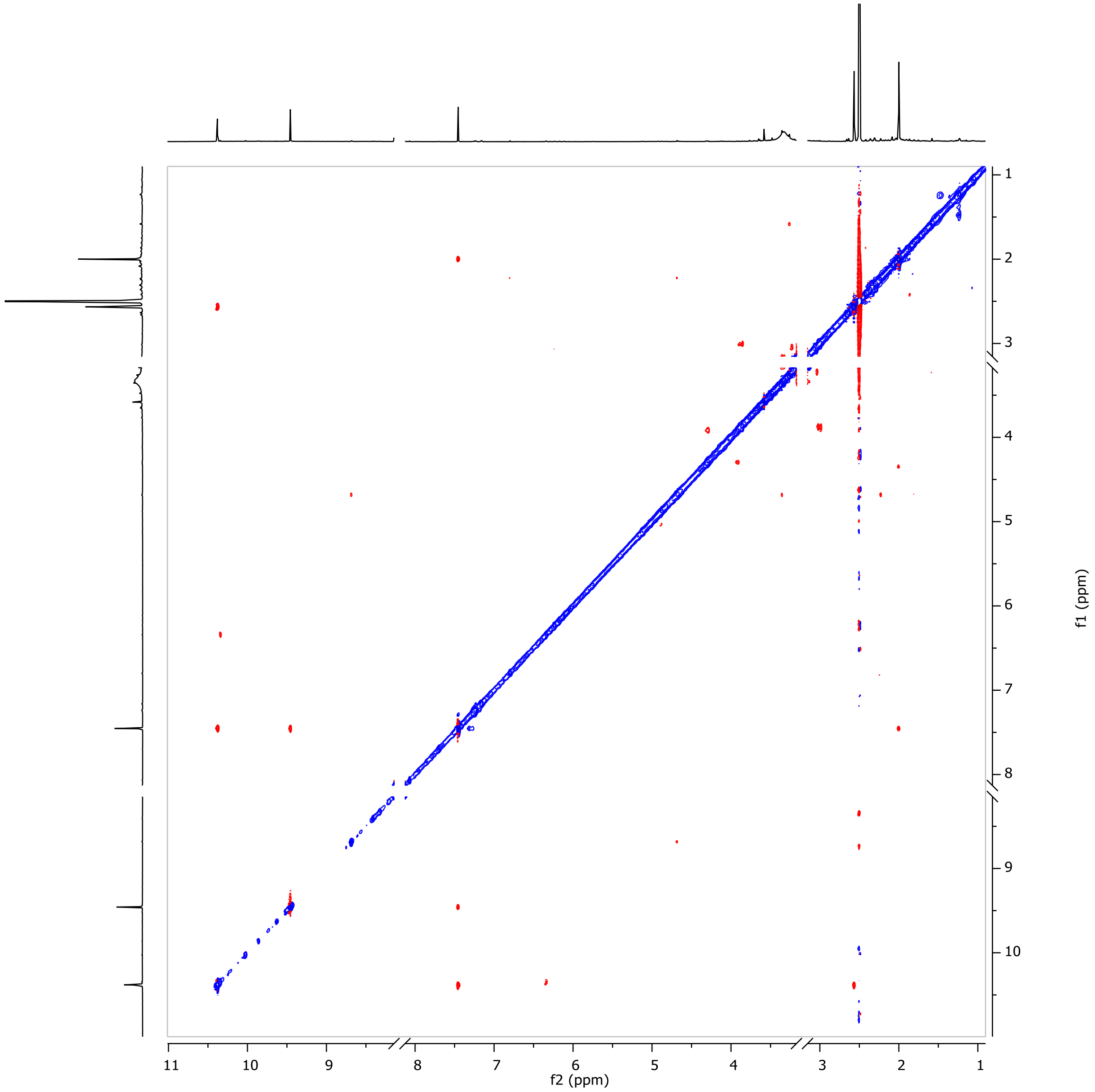

Figure S25. ROESY spectrum of **4** in DMSO-d_6 at 500 MHz.

Generic Display Report

Analysis Info

Analysis Name	C:\SEL22\Amazon\ANN-B-2\A10.H20.2.3_GA3_01_42641.d	Acquisition Date	27.10.2022 22:39:42
Method	42641.m	Operator	esu
Sample Name	A10.H20.2.3	Instrument	amaZon speed
Comment			

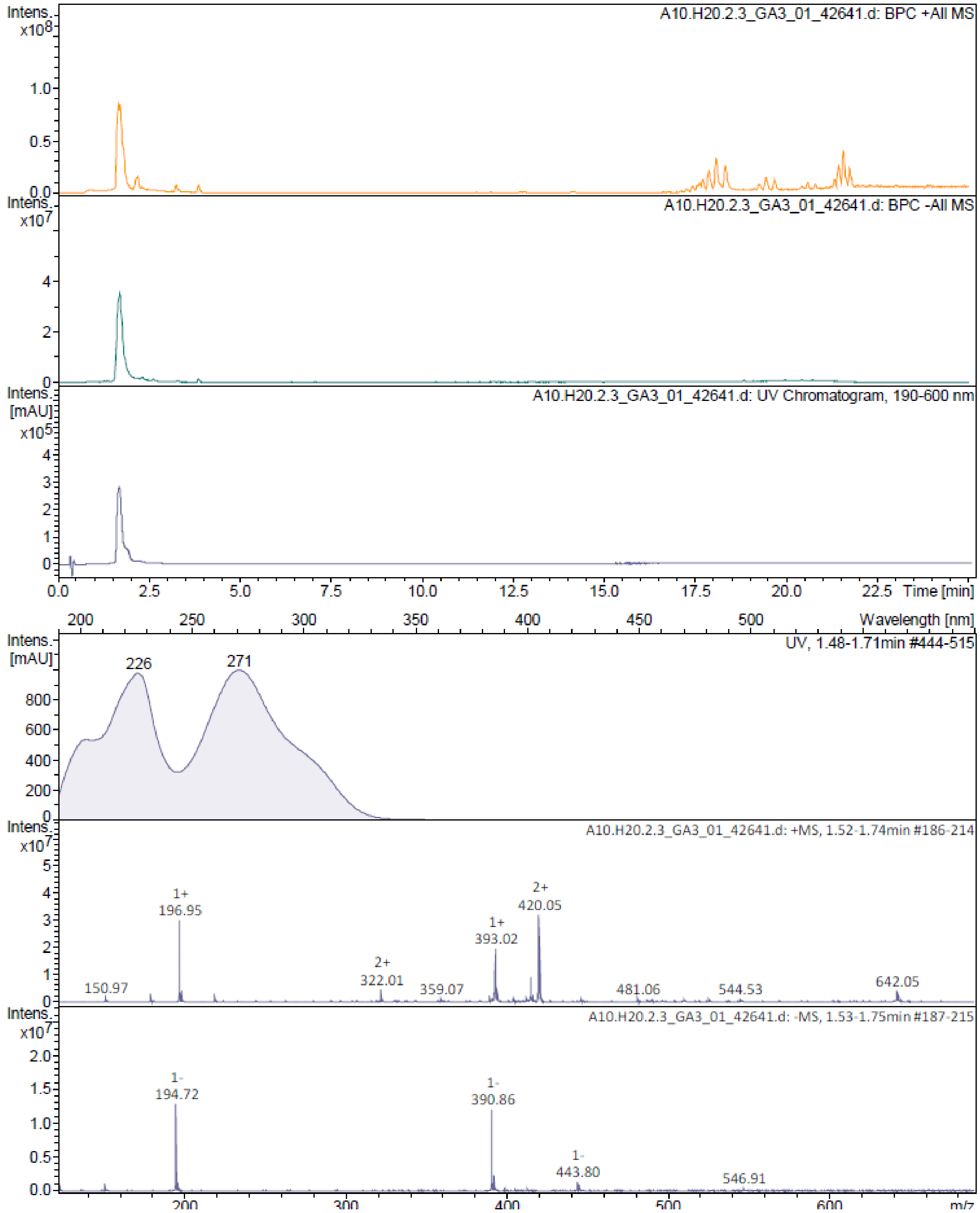

Bruker Compass DataAnalysis 4.4 printed: 07.11.2022 11:56:06 by: sel22 Page 1 of 1

Figure S26. HPLC chromatogram, UV and LR-ESI-MS spectra of **5**.

Generic Display Report

Analysis Info Acquisition Date 11.11.2022 15:34:21

Analysis Name	S:\DATA\MaXis\sel22_SherifElsayed\22_11\A10.H2O.2.3_46_01_10998.d		
Method	pos_säure_10000_screening_ms_100_2500_line.m	Operator	ate06
Sample Name	A10.H2O.2.3	Instrument	maXis
Comment	Screening01<br>Waters Acquity UPLC BEH C_18 1,7um 2.1x50mm		

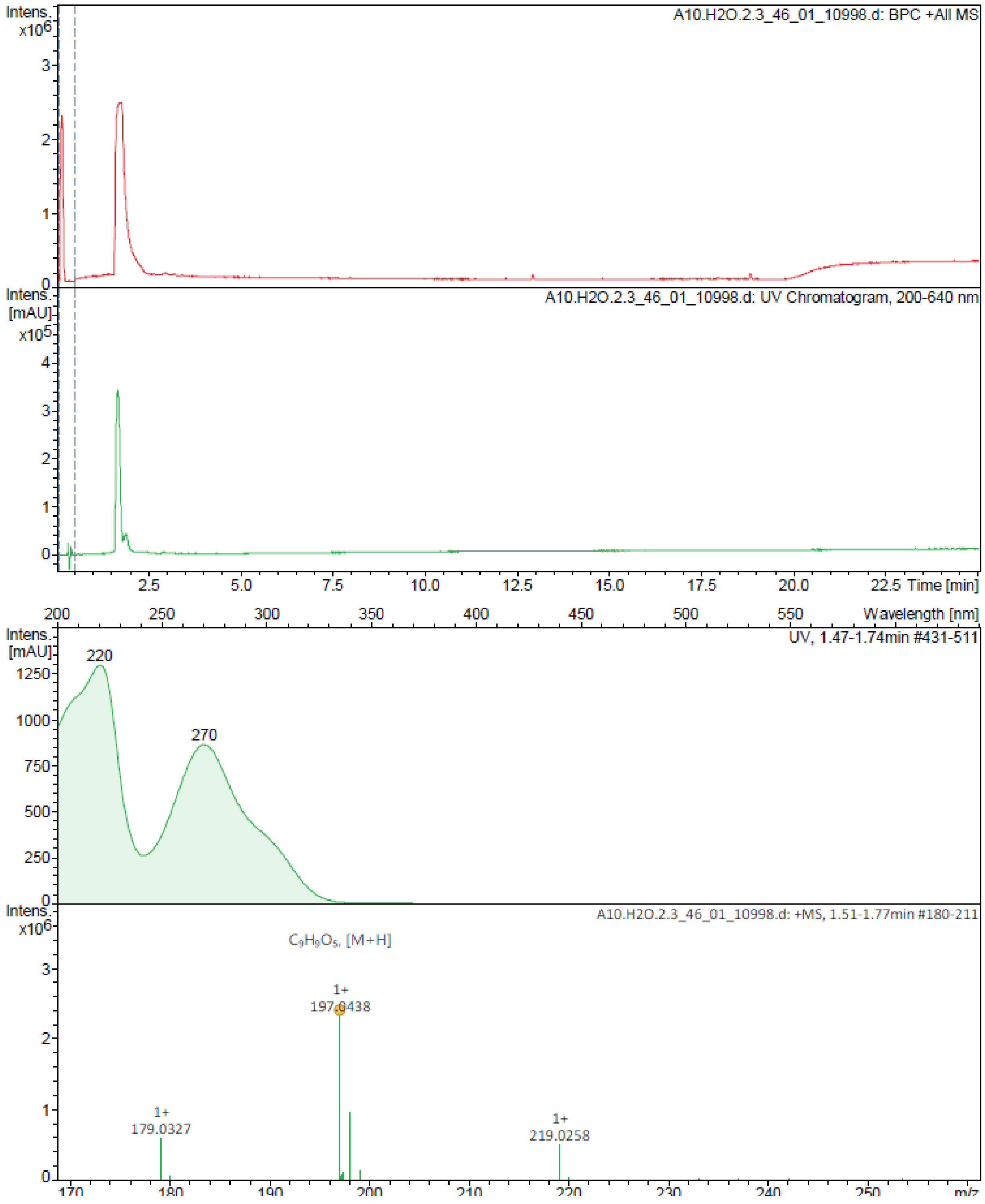

Bruker Compass DataAnalysis 4.4 printed: 14.11.2022 11:14:37 by: sel22 Page 1 of 1

Figure S27. HPLC chromatogram, UV and HR-ESI-MS spectra of **5**.

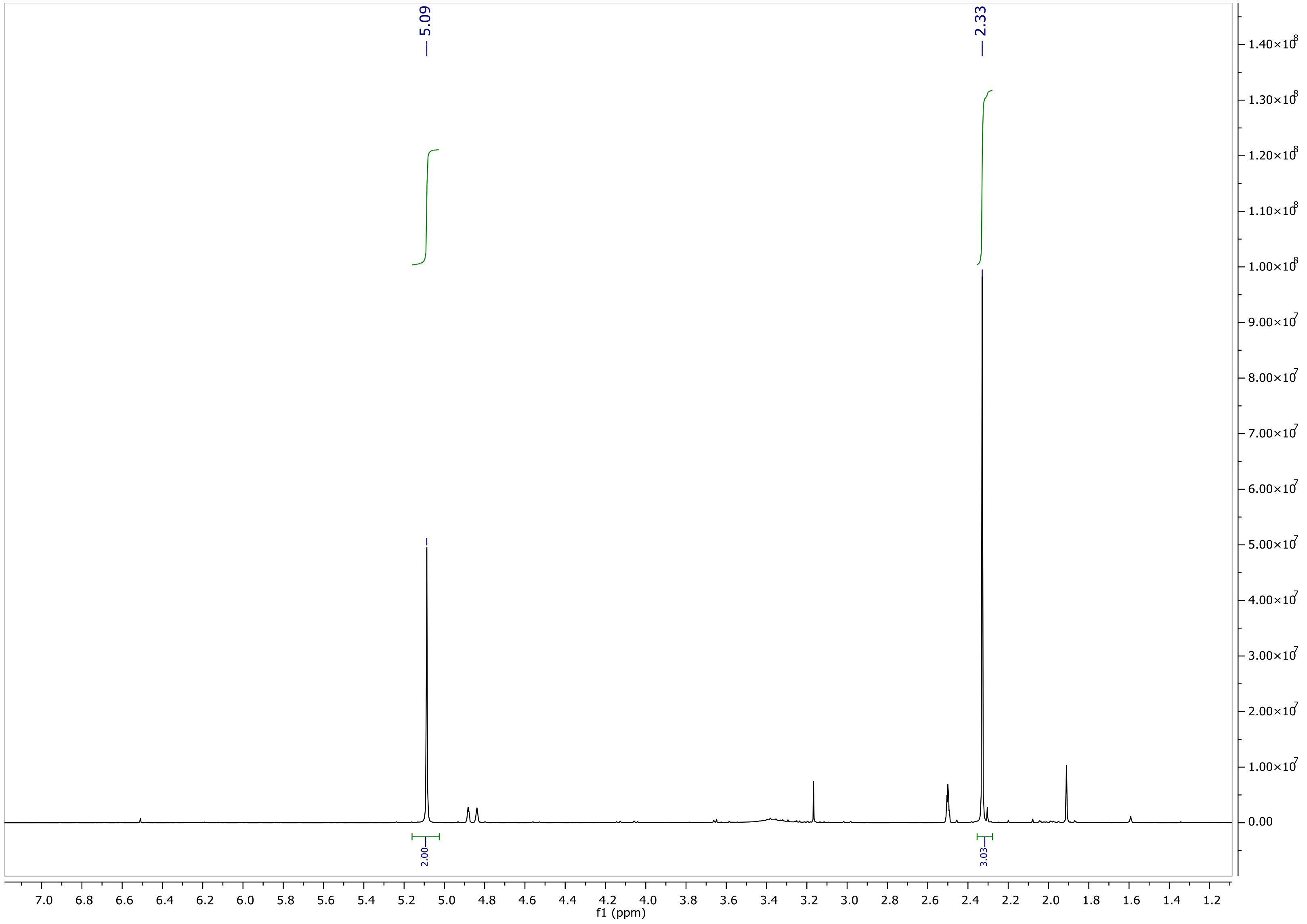

Figure S28. ^{1}H NMR spectrum of **5** in DMSO-d_6 at 500 MHz.

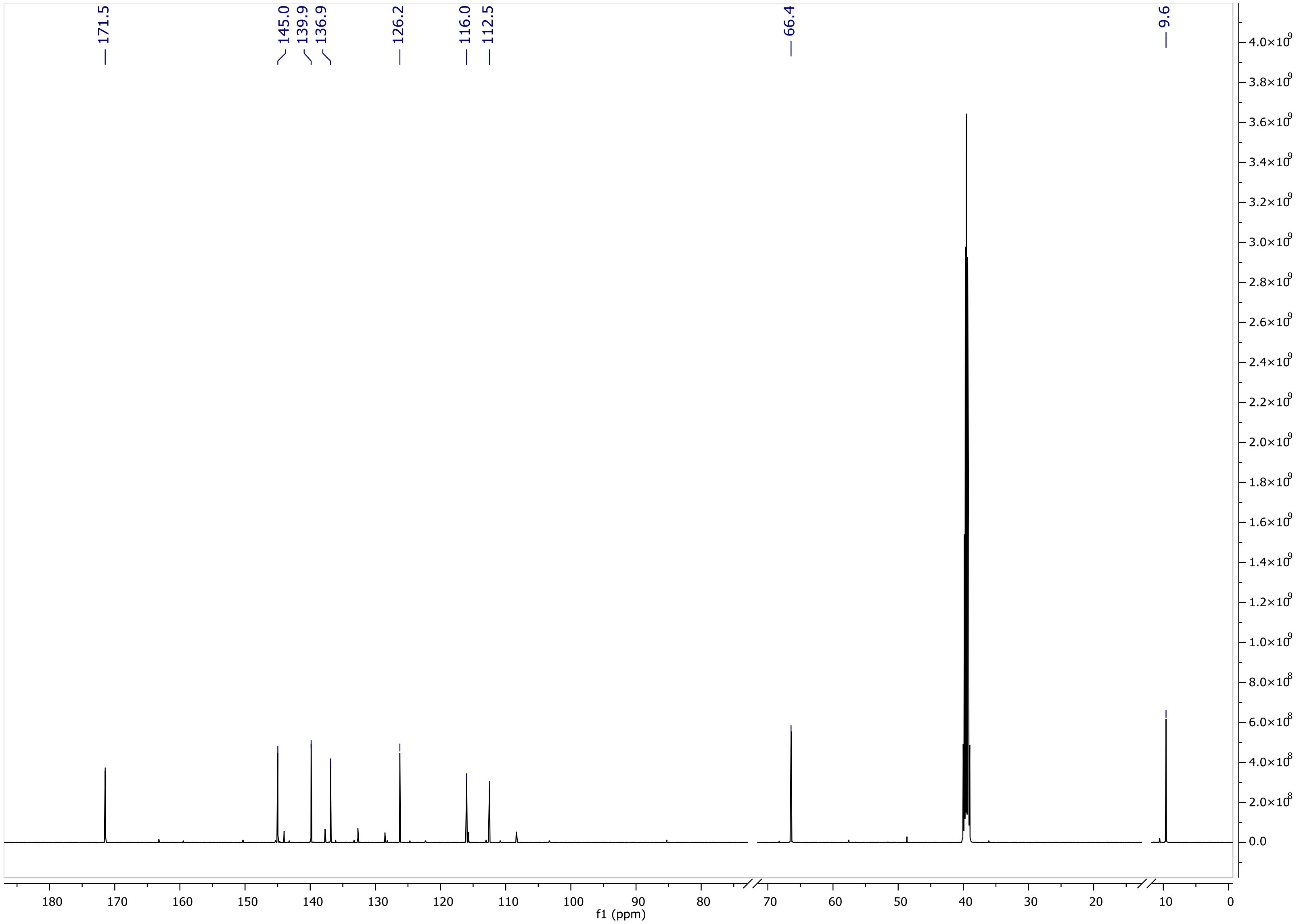

Figure S29. ^{13}C NMR spectrum of **5** in DMSO-d_6 at 125 MHz.

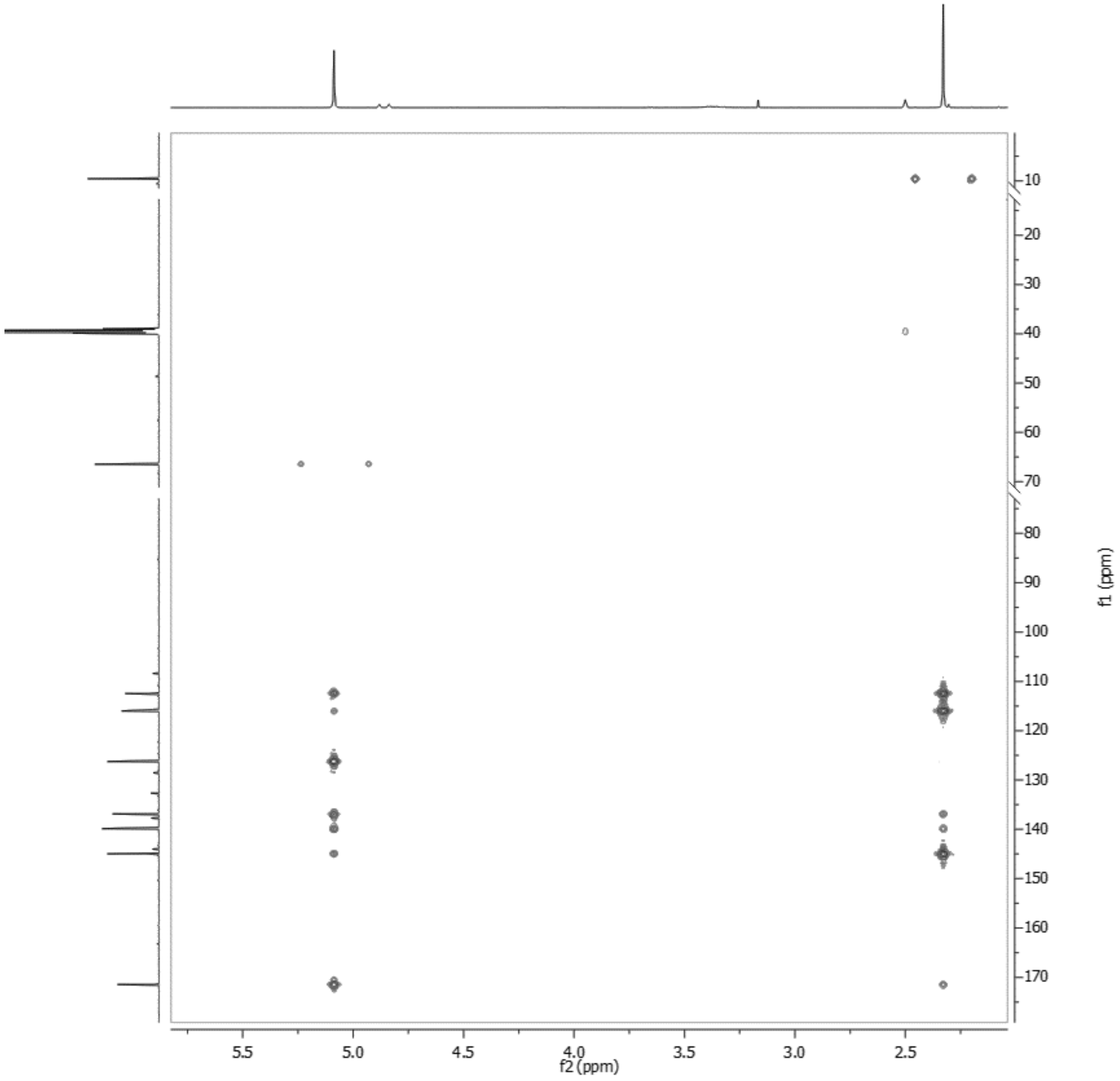

Figure S30. HMBC spectrum of **5** in DMSO-d_6 at 500 MHz.

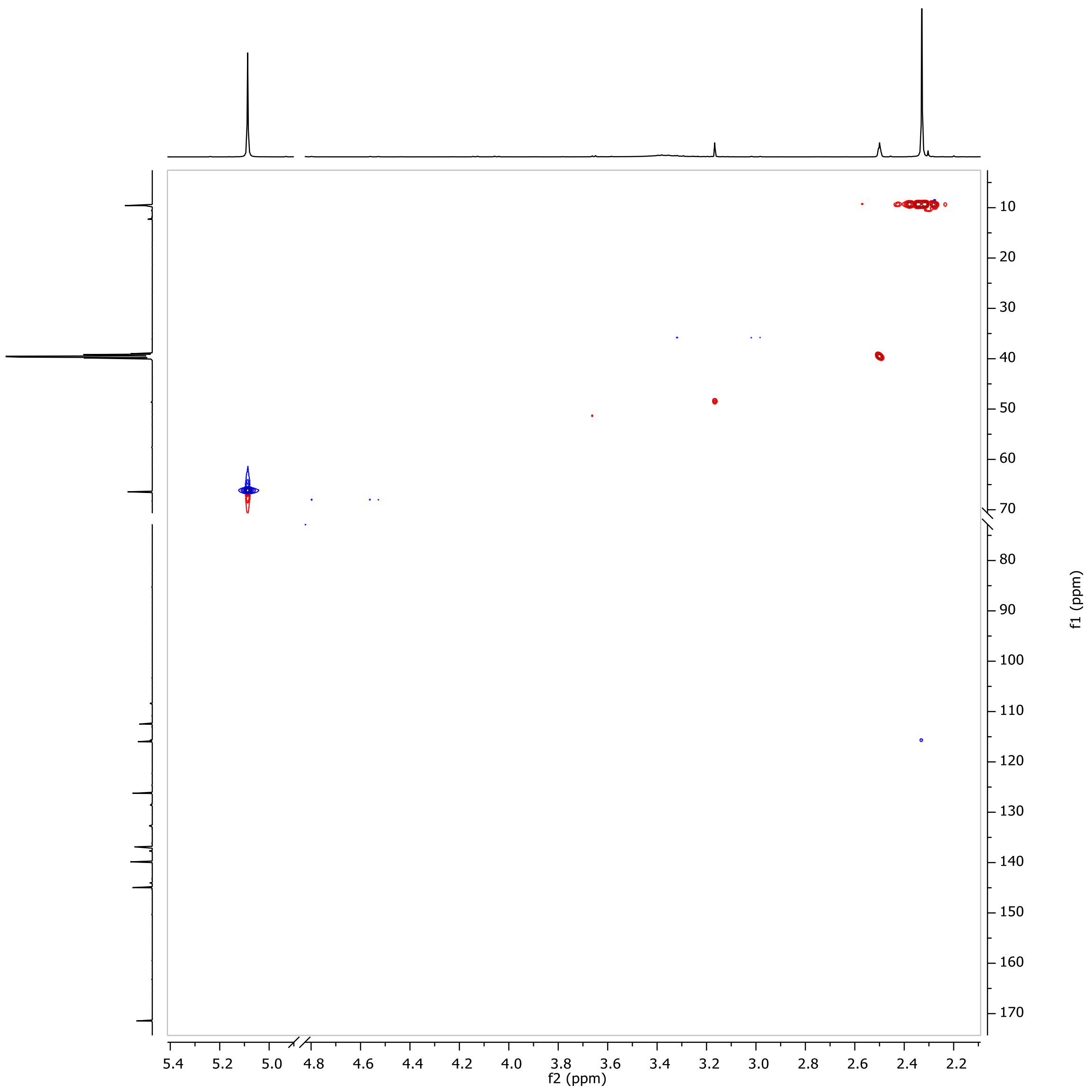

Figure S31. HSQC spectrum of **5** in DMSO-d_6 at 500 MHz.

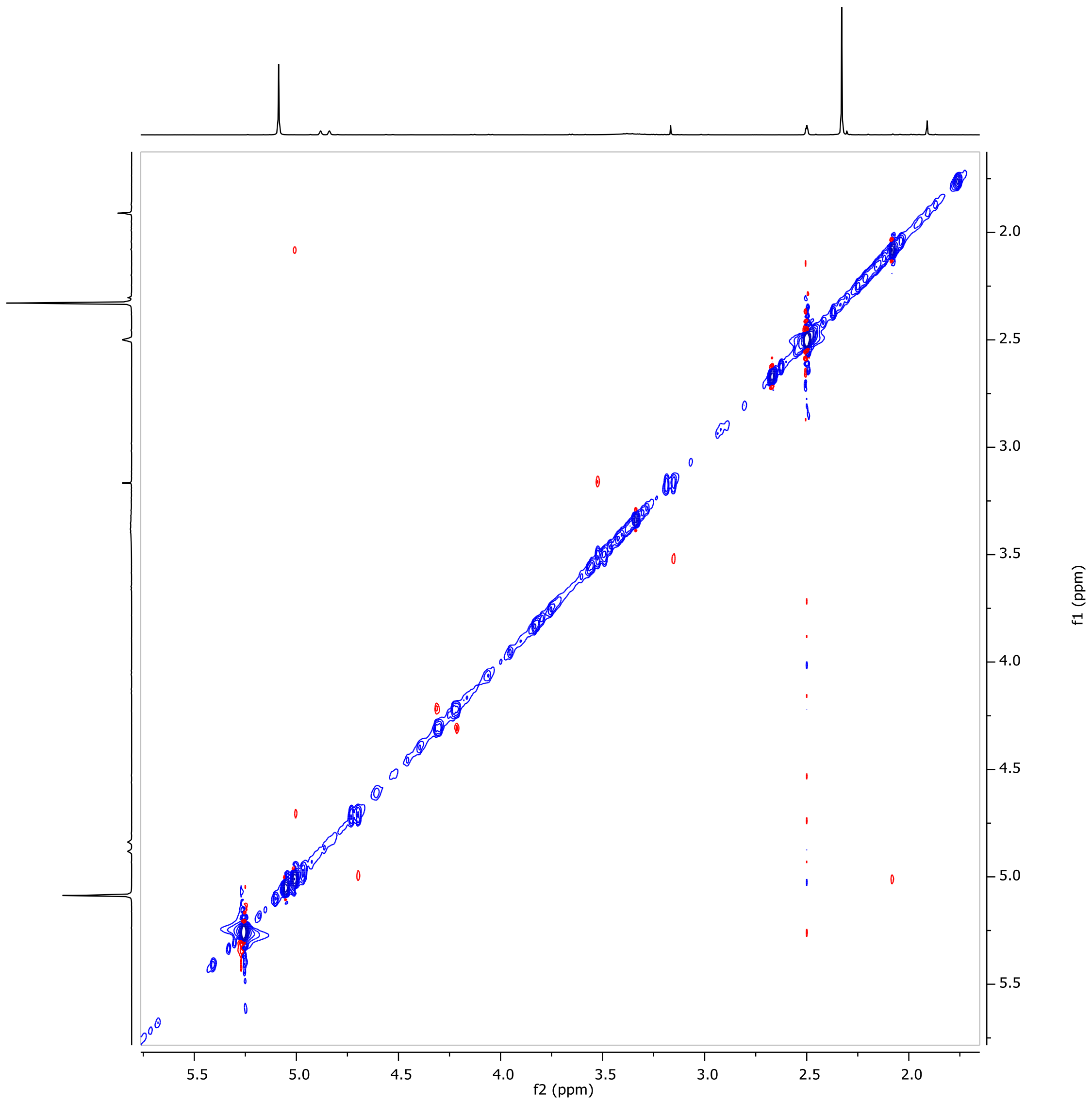

Figure S32. ROESY spectrum of **5** in DMSO-d_6 at 500 MHz.

Generic Display Report

Analysis Info

Analysis Name	C:\SEL22\Amazon\ANN-B-2\A10.H20.2.2_GA2_01_42640.d	Acquisition Date	27.10.2022 22:03:33
Method	42640.m	Operator	esu
Sample Name	A10.H20.2.2	Instrument	amaZon speed
Comment			

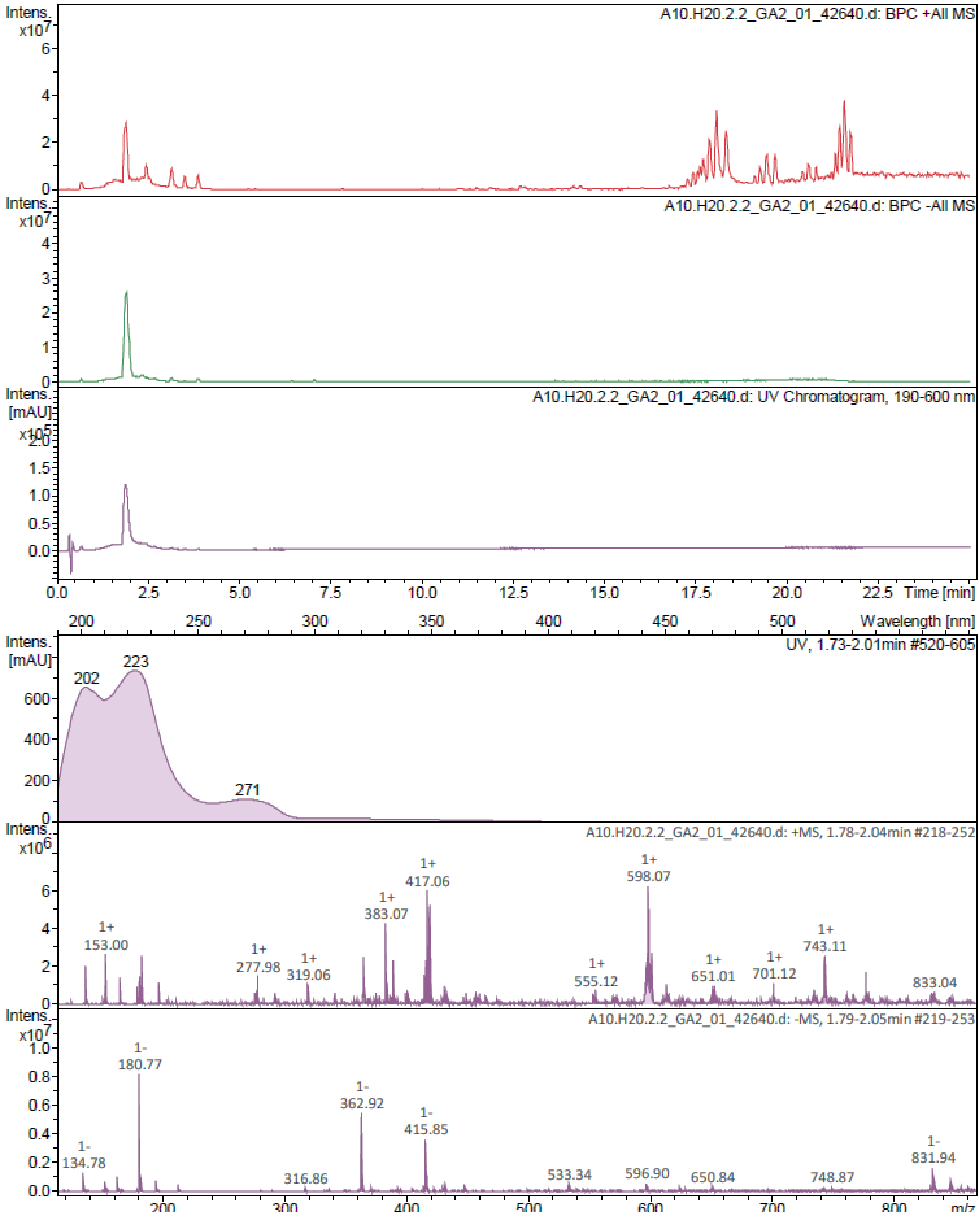

Bruker Compass DataAnalysis 4.4 printed: 07.11.2022 11:55:12 by: sel22 Page 1 of 1

Figure S33. HPLC chromatogram, UV and LR-ESI-MS spectra of **6**.

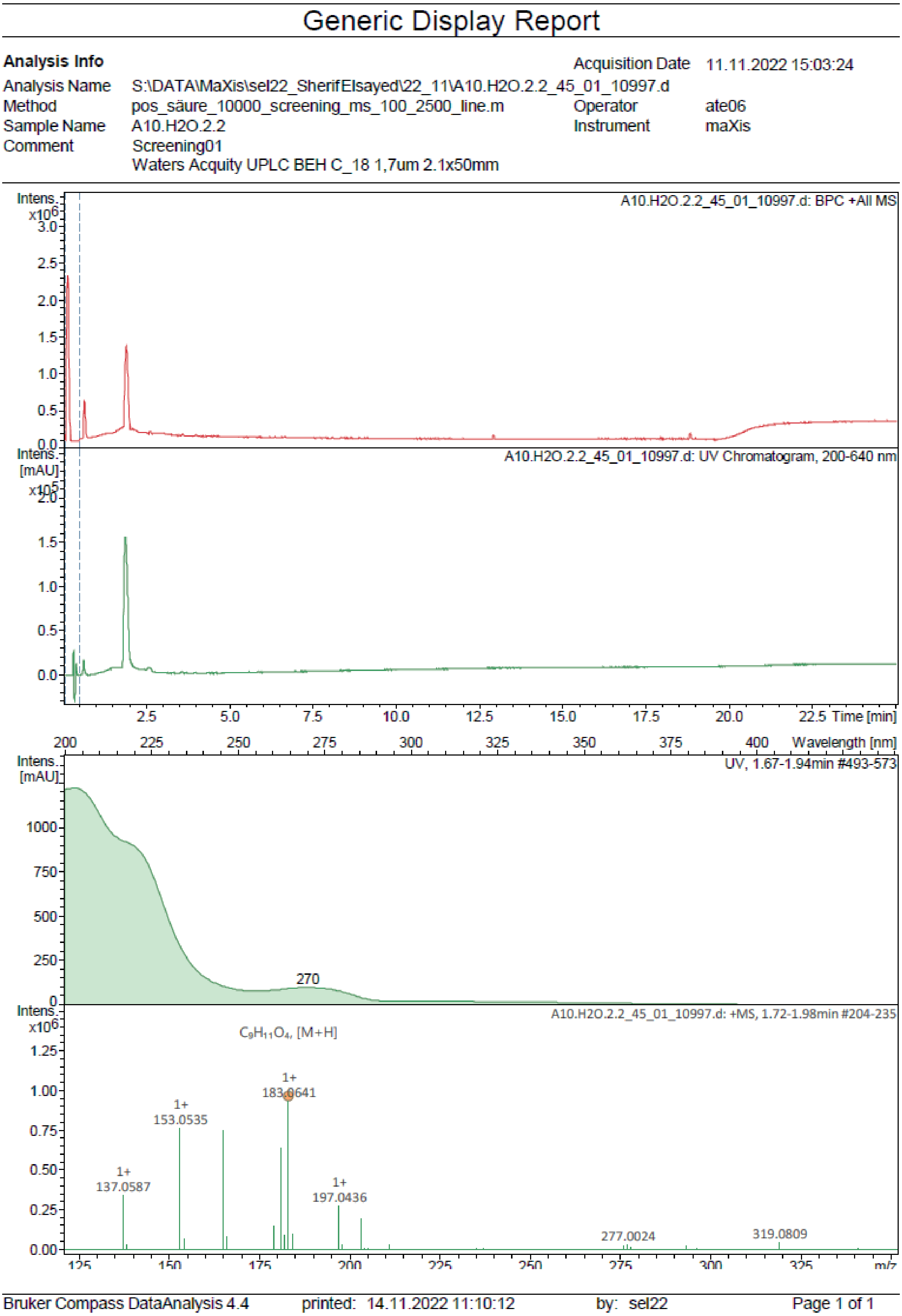

Figure S34. HPLC chromatogram, UV and HR-ESI-MS spectra of **6**.

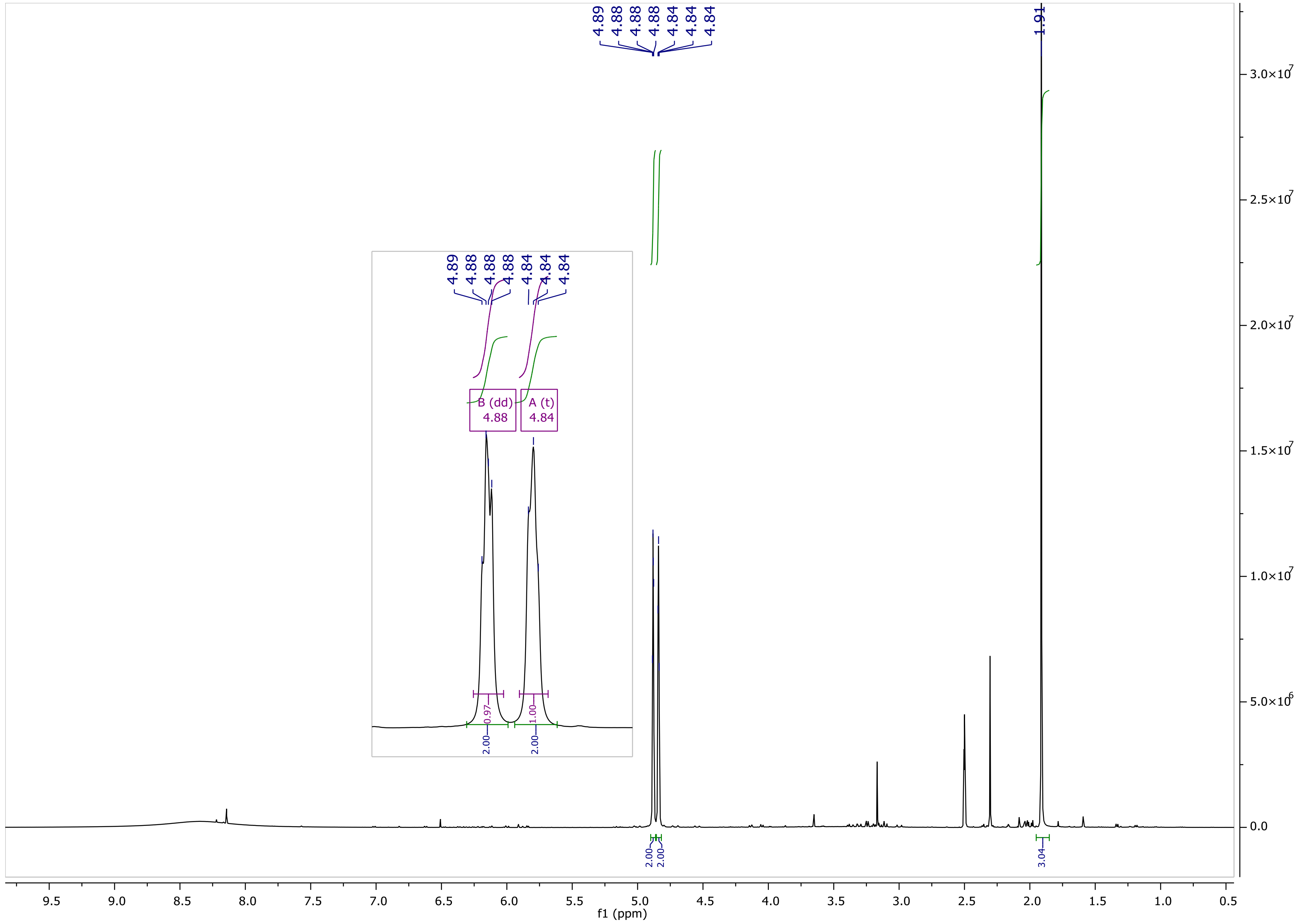

Figure S35. ^{1}H NMR spectrum of **6** in DMSO-d_6 at 500 MHz.

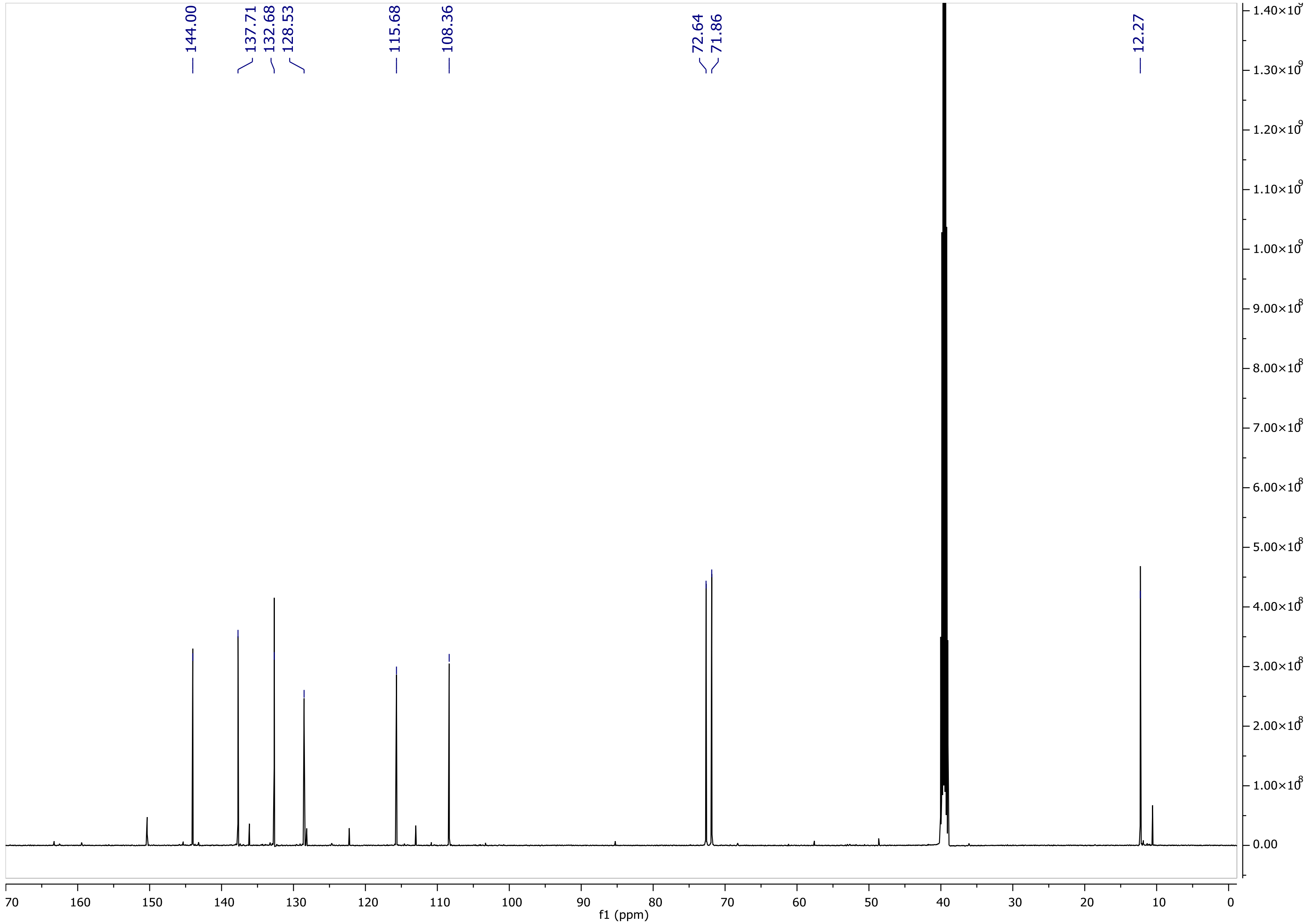

Figure S36. ^{13}C NMR spectrum of **6** in DMSO-d_6 at 125 MHz.

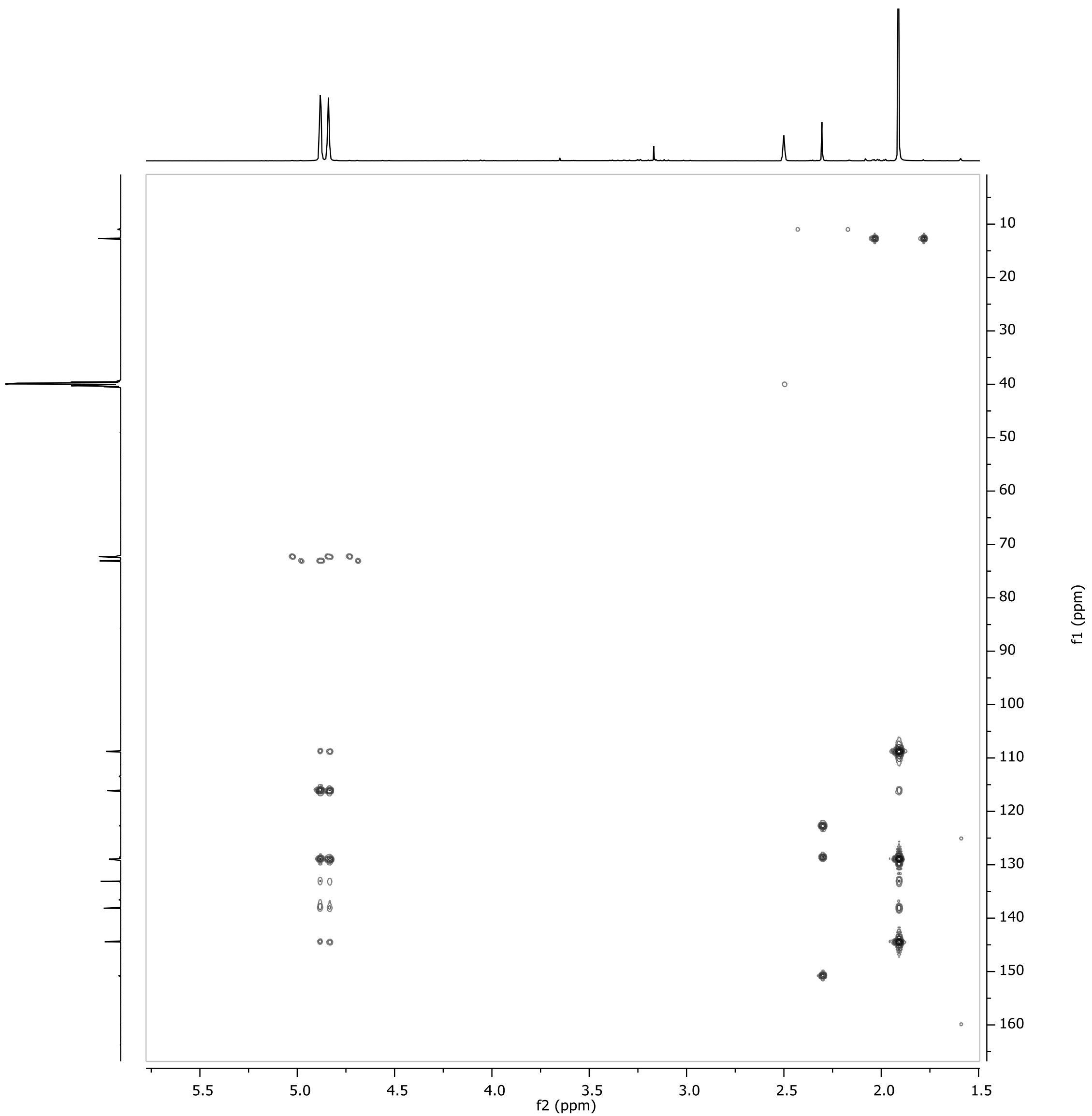

Figure S37. HMBC spectrum of **6** in DMSO-d_6 at 500 MHz.

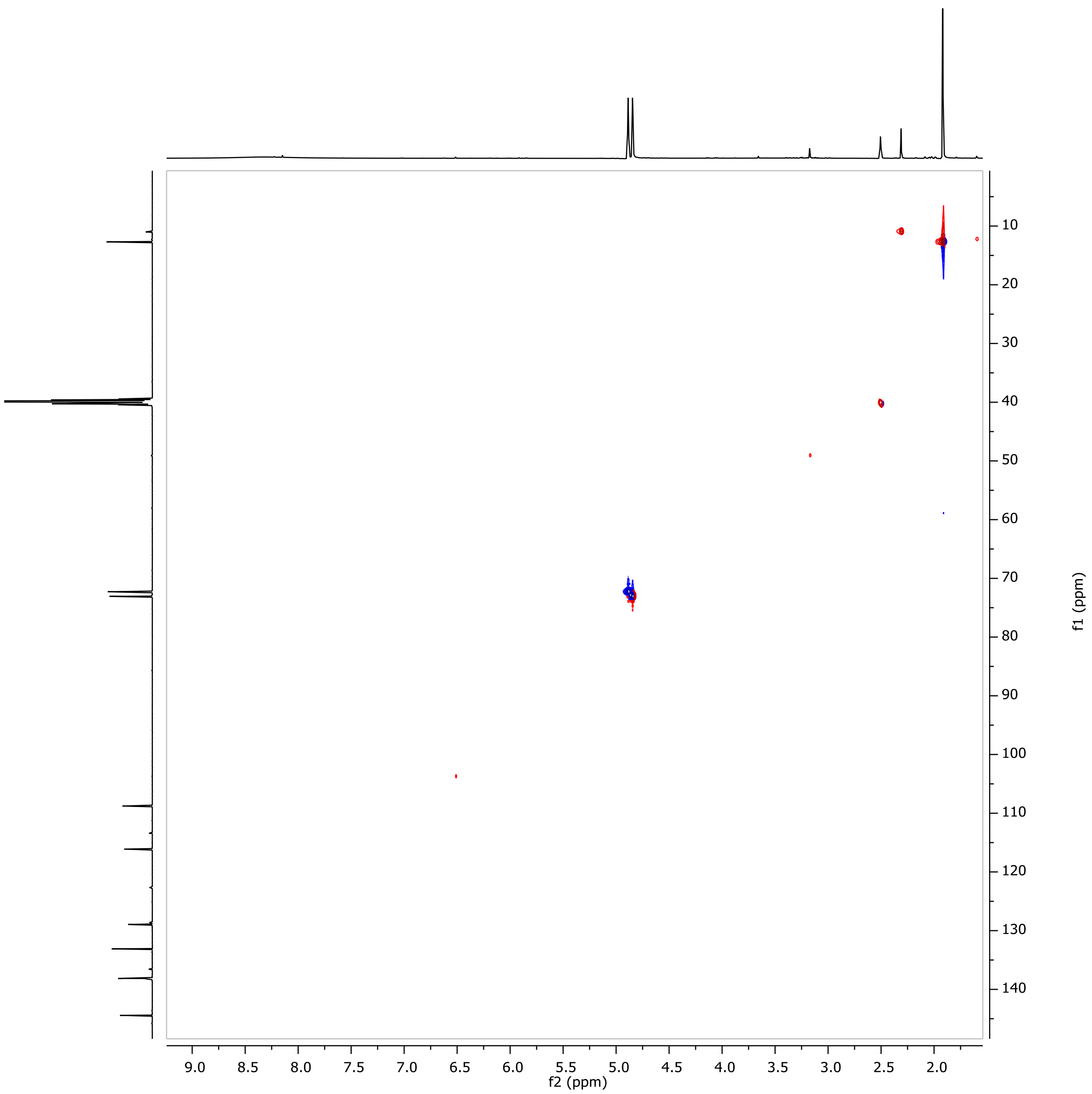

Figure S38. HSQC spectrum of **6** in DMSO-d_6 at 500 MHz.

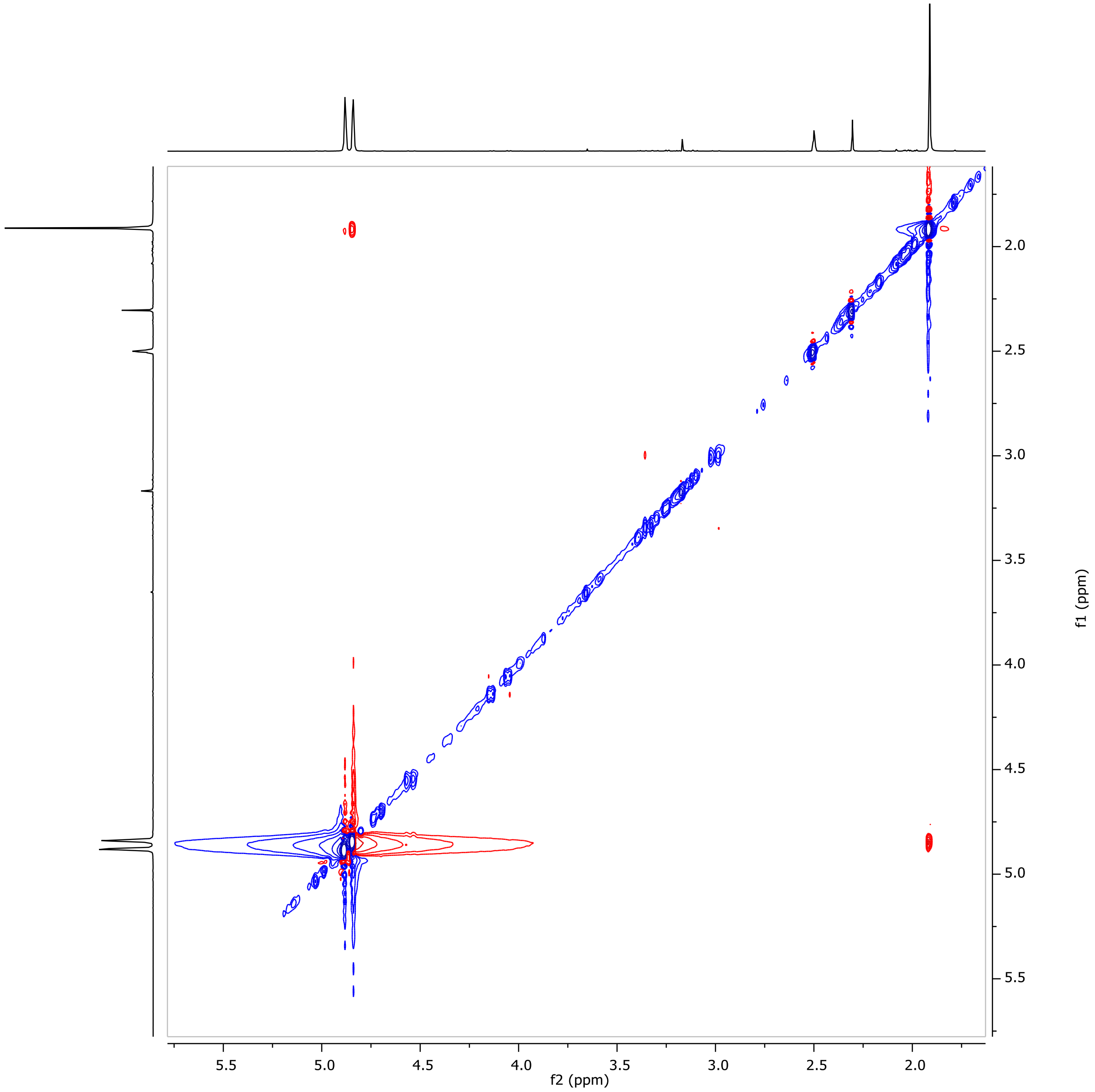

Figure S39. ROESY spectrum of **6** in DMSO-d_6 at 500 MHz.
